# ContextMap 2: fast and accurate context-based RNA-seq mapping

**DOI:** 10.1186/s12859-015-0557-5

**Published:** 2015-04-17

**Authors:** Thomas Bonfert, Evelyn Kirner, Gergely Csaba, Ralf Zimmer, Caroline C Friedel

**Affiliations:** Institute for Informatics, Ludwig-Maximilians-Universität München, Amalienstr. 17, Munich, 80333 Germany

## Abstract

**Background:**

Mapping of short sequencing reads is a crucial step in the analysis of RNA sequencing (RNA-seq) data. ContextMap is an RNA-seq mapping algorithm that uses a context-based approach to identify the best alignment for each read and allows parallel mapping against several reference genomes.

**Results:**

In this article, we present ContextMap 2, a new and improved version of ContextMap. Its key novel features are: (i) a plug-in structure that allows easily integrating novel short read alignment programs with improved accuracy and runtime; (ii) context-based identification of insertions and deletions (indels); (iii) mapping of reads spanning an arbitrary number of exons and indels. ContextMap 2 using Bowtie, Bowtie 2 or BWA was evaluated on both simulated and real-life data from the recently published RGASP study.

**Conclusions:**

We show that ContextMap 2 generally combines similar or higher recall compared to other state-of-the-art approaches with significantly higher precision in read placement and junction and indel prediction. Furthermore, runtime was significantly lower than for the best competing approaches. ContextMap 2 is freely available at http://www.bio.ifi.lmu.de/ContextMap.

**Electronic supplementary material:**

The online version of this article (doi:10.1186/s12859-015-0557-5) contains supplementary material, which is available to authorized users.

## Background

Sequencing of RNA using next generation sequencing technology (RNA-seq) has become the standard approach for analyzing the transcriptomic landscape of a cell [1,2]. The first step in RNA-seq data analysis generally consists in determining the transcriptomic origin of the sequenced reads (= read mapping) [3], i.e. the best alignment of each read against a transcript. Here, the major challenge results from the fact that even for well-annotated species not all transcripts, in particular rare or non-coding transcripts [4], are known. Thus, alignment against known transcript sequences using short read alignment programs such as Bowtie [5] cannot identify reads from novel transcripts, in particular spliced reads crossing novel exon-exon junctions. Unspliced reads, in contrast, are easily mapped using genome alignments.

Currently, many different RNA-seq mapping algorithms are available, such as TopHat [6], TopHat2 [7], or MapSplice [8] (see also [9] for an overview). In most cases, these approaches combine alignment against reference sequences (i.e. a genome and/or transcriptome) using short read aligners, such as Bowtie [5] or Bowtie 2 [10], with sophisticated strategies for identifying spliced reads crossing exon-exon junctions. A common strategy for this purpose involves splitting reads into smaller segments before aligning and is used e.g. by TopHat2 and MapSplice. Other mapping approaches, such as STAR [11] or GSNAP [12], use their own alignment methods to identify spliced reads without fragmenting read sequences.

Independent of the strategy for identifying spliced reads, existing RNA-seq mapping approaches were implemented to use only specific short read alignment programs, in most cases Bowtie. Thus, they cannot be easily extended to make use of novel developments in short read alignment, e.g. Bowtie 2 [10] or BWA [13], which improve alignment speed, recall or precision [14]. Furthermore, they generally identify the best alignment for each read based only on the number of mismatches and do not take into account information provided by alignments of other reads. We recently proposed a different approach, ContextMap, to identify the most likely mapping for a read based on all reads aligned to the same general location, the so-called context [15]. This approach also has the advantage that it allows parallel mapping against several reference genomes in a straightforward way [16].

In this article, we present ContextMap 2, an extension of the ContextMap strategy, which among other improvements addresses the problem of integrating different short read alignment programs. The key novel features of ContextMap 2 are:

(i) It provides an easy-to-use plug-in interface for integrating different short read alignment programs into the mapping workflow. This flexibility guarantees that ContextMap can be quickly adapted to newly developed read alignment algorithms.

(ii) It extensively uses local read alignment options of novel short read alignment programs such as Bowtie 2 or BWA to accurately detect spliced reads.

(iii) It precisely predicts the exact position of deletions or insertions (indels) by using the information provided by all reads in the same context.

We evaluated the performance of ContextMap 2 using Bowtie, Bowtie 2 and BWA as integrated alignment programs on both simulated and real-life RNA-seq data used by the RGASP consortium in a recent evaluation of RNA-seq mapping programs [17]. The comparison of ContextMap 2 to the best performers of this study showed that it combined high recall with high precision on read placement, splice junctions, multi-junction reads and indels. While individual competing RNA-seq mapping programs outperformed ContextMap 2 on some of these tasks, none was consistently better or performed comparably well in all of them. Furthermore, ContextMap 2 was generally at least twice as fast as the best competing methods.

## Implementation

### Overview of ContextMap 2

ContextMap 2 is based on the ContextMap approach for RNA-seq read mapping [15]. Here, the central concept is the so-called read context, which is defined as a set of reads all originating from the same stretch of the genome and likely corresponding to transcripts of the same or overlapping genes. The first implementation of ContextMap was focused on improving initial mappings provided by other RNA-seq mapping programs, but has more recently been extended into a standalone version that also allows parallel mapping against several reference genomes [16].

Similar to other mapping approaches, this first ContextMap implementation used a modified version of Bowtie for alignment. Thus, it suffered from the same problem as most state-of-the-art mapping approaches that newly developed short read alignment programs could not be easily integrated to replace the used Bowtie version. Furthermore, variable read lengths were not supported and reads crossing multiple exon-exon junctions or containing indels were not mapped. All of these problems are addressed by ContextMap 2.

**Figure 1:**
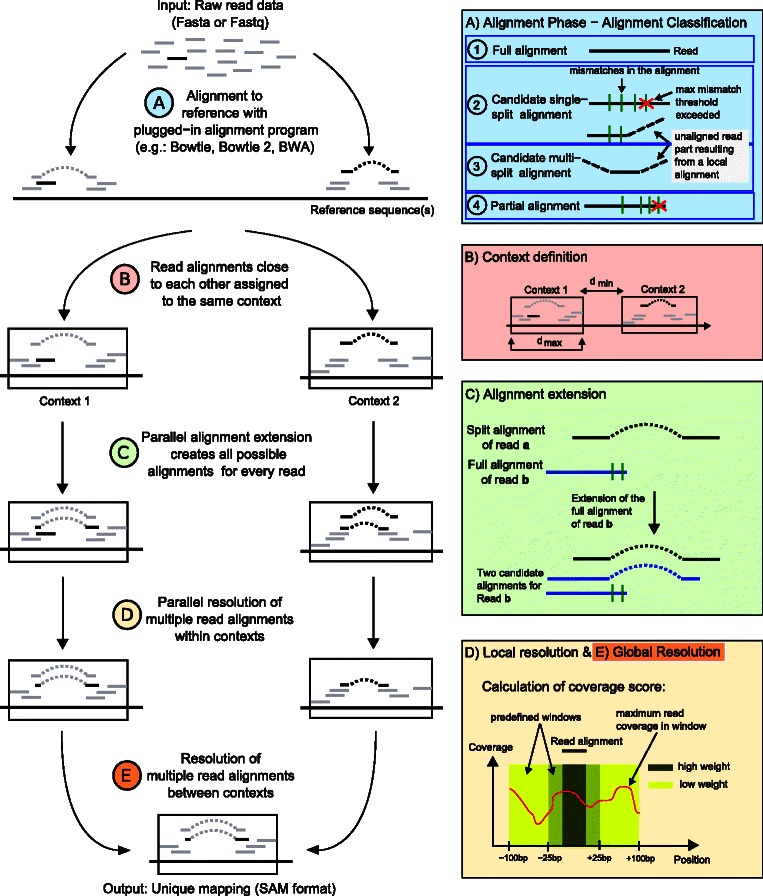
Workflow of ContextMap 2.**(A)** Reads are aligned to the reference sequence(s) using the integrated short read alignment program and the resulting alignments are classified into 4 different categories (top box, right side: full alignment, candidate single-split alignment, candidate multi-split alignment, and partial alignment). Dashed lines indicate unaligned sequence parts resulting from local alignments. Candidate single- and multi-split alignments are extended to split alignments using the sliding window approach (Figure 2). **(B)** Alignments less than *d*_*min*_ apart are assigned to the same context. The maximum context size *d*_*max*_ can be defined by the user (default is the average length of a mammalian mRNA). **(C)** Alignment extension of full (green box) and split alignments (see Additional file 1: Supplementary methods) to determine all valid alignments for a read. **(D)** + **(E)** Resolution of the best alignment for each read first within each context (D, local resolution) and then between all contexts (E, global resolution). For this purpose, a support score is calculated based on closely located alignments of other reads (bottom box, right side, and Additional file 1: Supplementary methods).

In the following, an overview of the five steps of the ContextMap 2 workflow is presented (see Figure 1). The details of each step are described following this overview and in Additional file 1: Supplementary methods.

**Figure 2:**
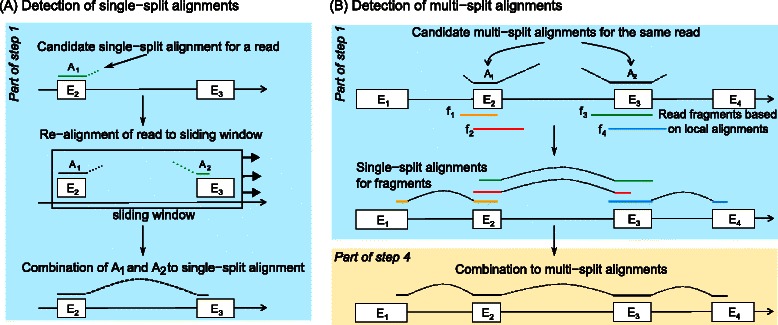
Detection of single-split and multi-split alignments in ContextMap 2.**(A)** Detection of single-split alignments as part of step 1. First, reads are aligned to the genome and candidate split alignments (*A*_1_) are identified. Second, reads with candidate split alignments are re-aligned within a window around the initial alignment to determine a completing alignment (*A*_2_). The use of smaller seed lengths than in the initial alignment allows recovering completing alignments shorter than the seed length used for the initial alignment. Finally, the alignments are combined to a complete split alignment. **(B)** Detection of multi-split alignments. For every candidate multi-split alignment, ContextMap 2 creates two fragments of the respective read sequence (i.e. *f*_1_ and *f*_2_ for *A*_1_ and *f*_3_ and *f*_4_ for *A*_2_). Subsequently, single-split alignments are detected for these fragments. Finally, overlaps of single-split alignments are combined to obtain a complete multi-split alignment after first identifying the best splice site for each split alignment as part of the resolution of overlapping splice sites in step 4 of ContextMap 2.

#### Step 1: Determination of initial alignments

This step includes both the determination of ungapped read alignments against one or several genomes using the integrated short read alignment program, e.g. BWA, as well as the extension of these alignments to alignments containing a splice junction (= split read alignments, see Figure 1A). For this purpose, ContextMap 2 first performs a seeded alignment of all reads against the reference sequences with user defined seed values of 20-30 nt. Here, ContextMap 2 can use programs that determine only end-to-end alignments (e.g. Bowtie) as well as programs that also determine local alignments (e.g. Bowtie 2 and BWA). An end-to-end alignment starts at the read start and ends with the read end. In contrast, a local alignment allows unaligned prefixes or suffixes of the read if this improves the alignment score. In case an alignment program allows alignment of the seed only at the start of the read, such as Bowtie, a “backward” alignment with the reversed read is also performed for reads for which no alignment beginning at the read start could be found.

Parameters of the underlying alignment program are set such that all alignments for which the seed can be aligned are retained, allowing for multiple alignments of each read. The resulting alignments are then classified into four categories:

1. *Full alignment:* if the read could be aligned end-to-end to the genome with a maximum number of mismatches (defined by the user).
2. *Candidate single-split alignment:* if the seed could be aligned at the start or end of the read, the end-to-end alignment of the read contains more than the allowed number of mismatches and the last allowed mismatch is at least a predefined distance from the end of the alignment. If the integrated short read alignment program also produces local alignments, unaligned read positions are counted as mismatches for this classification.
3. *Partial alignment:* if the same criteria apply as in (2) but the last allowed mismatch is less than the predefined distance from the alignment end.
4. *Candidate multi-split alignment:* if only a local alignment could be determined with both a prefix and suffix of the read unaligned.

Following this classification, candidate single-split and multi-split alignments are extended to complete split alignments as described further below.

#### Step 2: Context definition

The alignments identified in the previous step are used to define contexts. For this purpose, read alignments are clustered into a context if their start or end positions on the genome are at most a maximum distance apart (Figure 1B). Contexts are treated independently of each other until step 5. This allows both mapping read sequences against several reference genomes, e.g. of the human host and infecting pathogens [16], as well as efficient parallelization of steps 3 and 4. Here, multiple alignments of each read to the same context or different contexts are allowed, which will be resolved in steps 4 and 5.

#### Step 3: Alignment extension

Once contexts have been defined, additional alignments are determined for each read based on the alignments found in the first step (Figure 1C). This alignment extension is performed in parallel for different contexts. Its objective is to identify all valid alignments for each read with a maximum number of mismatches, such that the best supported alignment can be selected in the subsequent steps.

For this purpose, full and partial read alignments are checked for an overlap with split alignments of other reads. If overlaps are found, additional split alignments are created for the corresponding reads using the splice junctions indicated by the overlapping split alignments. Furthermore, all possible split alignments are generated for each read for which at least one split alignment was identified in step 1 (see Additional file 1: Supplementary methods for details). In both cases, only alignments are used that do not exceed the maximum mismatch criterion. At the end of this step, several different alignments have been created for each read, resulting in multiple alignments both within and between contexts.

#### Step 4: Local resolution of alignments within contexts

In this step, the best alignment for each read is determined within each context by taking other read alignments into account (Figure 1D). For this purpose, the three best supported splice sites among overlapping splice sites are determined first. For this purpose, the following evidence score for each splice site is used:

(1) $$ evidence = \sum\limits_{i=0}^{m} \left(w^{i} \cdot n_{i}\right)  $$

Here, *n*_*i*_ is the number of reads (full, split or partial) with *i* mismatches supporting the splice site, *m* the maximum number of mismatches allowed and *w* a value <1 (default *w*=0.3) (see Additional file 1: Supplementary methods for details).

Split read alignments not using any of the best supported splice sites are discarded. Subsequently, a support score is calculated for the remaining read alignments based on the number of reads aligned within and around the read alignment. In principle, the support score is a weighted sum of maximum read coverages in predefined windows around the read alignment (see Additional file 1: Supplementary methods for details). Among several alternative alignments for the same read within each context, the one with the largest support score is then chosen.

#### Step 5: Global resolution of alignments between contexts

In this final step, multiple read alignments to several different contexts are resolved as in step 4 after recalculating support scores based on the read alignments chosen for each context (Figure 1E). Thus, at the end of each step, each read is aligned to only one position in (at most) one context. If more than one reference sequence was provided, this will also automatically result in the choice of one reference sequence of origin for each read.

### Plug-in structure of ContextMap 2

ContextMap 2 provides a plug-in interface which allows integrating any short read alignment program without modification if it meets the following requirements:

1. The alignment program has to support seeded alignments with adjustable seed lengths to allow use of different seed lengths in different steps of ContextMap 2.
2. The alignment program has to provide a tool to prepare an index of any reference sequence. Indexing reference sequences is a common strategy of all state-of-the-art short read alignment programs to speed up alignment.
3. If the read alignment program includes an option to identify indels, it must be possible to deactivate this option. ContextMap 2 uses its own context-based strategy for predicting the exact position of indels.
4. The output has to be in SAM format [18].

The interface for plugging in a short read alignment program into ContextMap 2 is composed of three methods, two for performing alignments at different steps of ContextMap 2 and one for indexing reference sequences.

Implementing the interface requires implementing methods for managing the external program calls. In addition, the alignment methods have to collect the determined alignments. For this purpose, two classes can be reused that perform these tasks for Bowtie, Bowtie 2 and BWA, which have already been integrated in ContextMap 2.

### Detection of single-split alignments

As part of step 1, ContextMap 2 extends candidate split alignments to single-split alignments, i.e. alignments crossing one exon-exon junction only, using a so-called sliding window approach (Figure 2A). This sliding window approach works in the following way: The sliding window is initiated at the left-most candidate split alignment on a chromosome and is extended to contain any overlapping alignment until a pre-defined maximum window length is exceeded. All candidate single-split alignments within this window are then extended to complete split alignments as described below. Afterwards, the current window is discarded and the next window is determined starting at the next candidate split alignment not completely contained in the previous window. This is repeated until all candidate split-alignments have been processed.

To determine the complete split alignments within each window, an index is built for the used short read alignment program covering the part of the reference sequence within the current window. This sequence is extended by *x* nucleotides (*x*= average intron size, can be defined by the user) downstream of the window if a candidate split alignment with the seed at the read start ends too close to the window end (i.e. the distance is less than the average intron size *x*). This allows finding split alignments that start within the window but end downstream of the window end. Similarly, an upstream sequence is added to the index if a candidate split alignment with the seed at the read end begins too close to the window start.

Using this dynamically built index and the corresponding short read alignment program, completing alignments of the unaligned read part are determined for each candidate split alignment within the sliding window (Figure 2A). This restricts the search space to a region covering only one or very few genes, allowing the use of smaller seed lengths of 10-15 nt. Since the window is very small and only a relatively small number of reads is covered by the window, this step is very fast. The original candidate split alignment and the completing alignment for each read are then combined into one split alignment and included in the set of initial alignments in addition to the full and partial alignments.

### Detection of multi-split alignments

The detection of multi-split alignments, i.e. alignments crossing more than one exon-exon junction, is a novel feature of ContextMap 2. It is based on local alignment options of recently developed alignment programs such as Bowtie 2 or BWA. Essentially, the local alignments are used to fragment the reads into smaller segments for which single-split alignments are then determined (see Figure 2B). In contrast to other approaches that fragment all reads into smaller equal-sized segments, only reads for which a local alignment was determined, i.e. candidate multi-split alignments, are fragmented by ContextMap 2.

For this purpose, candidate multi-split alignments (= local alignments with suffix and prefix of the read not aligned) to the same genomic region are collected using the same sliding window approach used for the single-split alignment detection. In fact, ContextMap uses a single run of the sliding window approach to process single- as well as multi-split alignments.

For each candidate multi-split alignment in the current sliding window, two fragments of the read sequence are generated. If read *r*=*r*_1_…*r*_*l*_ (*l*= read length) has been aligned at positions *r*_*i*_…*r*_*j*_, the first fragment consists of the subsequence *f*_1_=*r*_*i*−*e*_…*r*_*i*−1_ *r*_*i*_…*r*_*j*_, where *e* is the predefined minimum exon size (default 20 nt). If the unaligned prefix (*r*_1_…*r*_*i*−1_) of the read is smaller than the minimum exon size *e*, *f*_1_=*r*_1_…*r*_*j*_. Similarly, the second fragment is defined as *f*_2_=*r*_*i*_…*r*_*j*_ *r*_*j*+1_…*r*_*j*+*e*_. If the unaligned suffix of the read (*r*_*j*+1_…*r*_*l*_) is shorter than *e*, *f*_2_=*r*_*i*_…*r*_*l*_.

The original local alignment then provides candidate split alignments for *f*_1_ and *f*_2_. The completing alignments to these candidate split alignments are found within the sliding window as described in the previous section. This results in single-split alignments for the fragments, which are added to the list of initial alignments determined in step 1 of ContextMap 2 and extended to all valid single-split alignments of the fragments in step 3.

The complete multi-split alignment of the whole read is determined in step 4 by merging overlaps of the single-split alignments for fragments of the same read after the resolution of pairwise overlapping splice sites. Thus, the precise location of the splice sites is first determined for the single-split alignments of the fragments before combining them to the complete multi-split alignments.

### Detection of indels

**Figure 3:**
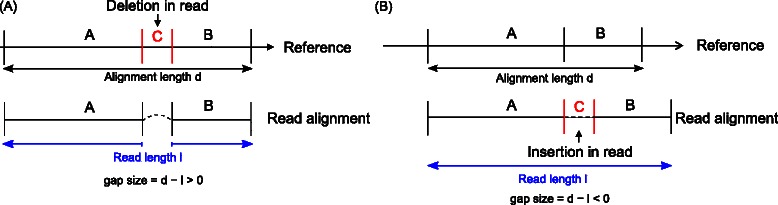
Deletions and insertions in reads as special cases of spliced reads.**(A)** Example of a read with a deletion compared to the reference sequence. In this case, the alignment length *d* is larger than the read length *l* and the gap size is positive. **(B)** Example of a read with an insertion compared to the reference sequence. Here, the alignment length *d* on the reference sequence is smaller than the read length *l* and the gap size is negative.

Essentially, the prediction of reads containing a deletion to the reference is the same as detecting spliced reads with a very small intron size (see Figure 3A). Similarly, a read containing an insertion to the reference can be considered as a special case of a spliced read spanning an intron with negative length (see Figure 3B). Thus, detection of deletions and insertions could be incorporated seamlessly into the single- and multi-split alignment detection procedure of ContextMap 2 by allowing both small and negative intron lengths, respectively. Conveniently, this also allows the mapping of reads containing both indels and splice sites by finding the corresponding multi-split alignment.

The distinction between indels and splice sites is only applied when preparing the output at the very end of the ContextMap 2 run. At this point, the gap size is determined for each split position in a single- or multi-split alignment (see Figure 3). The gap size is defined as *d*−*l*, where *d* is the alignment length on the reference genome and *l* is the read length. If the gap size is negative and its absolute value at most a user defined maximum insertion size (default = 10 nt), this split position is classified as an insertion. If the gap size is between 1 and a user defined maximum deletion size (default = 10 nt), it is classified as a deletion. If the gap size is between a user defined minimum intron size (default = 50 nt) and a user defined maximum intron size (default = 300,000 nt), the split is classified as an intron. Split alignments with gap sizes that do not fall into these ranges are not determined when detecting single- and multi-split alignments.

## Results and discussion

### Data sets and methods for evaluation

Evaluation of ContextMap 2 was performed on simulated and real data previously used by the RGASP consortium for the systematic evaluation of RNA-seq mapping programs [17] (see Additional file 1: Table S1 for a summary). The simulated data was generated using the simulation program BEERS, which is provided with the RUM pipeline [19]. Two data sets were simulated, each containing 80 million 76-nucleotide paired-end reads (= 40 million read pairs). The second data set is more challenging than the first as higher rates of substitution errors, indel polymorphisms and reads from unannotated isoforms were simulated. The real data consists of RNA-seq data of the human K562 cell line (whole cell, cytoplasmic and nuclear fraction) from the ENCODE project [20] (2 replicates each, resulting in 6 samples). Each sample consisted of ∼200 million 76-nucleotide paired-end reads (∼100 million read pairs).

We compared ContextMap 2 against the best performing RNA-seq mapping approaches identified in the RGASP study. These included MapSplice [8], STAR [11], and GSNAP [12]. We also included TopHat [6] (denoted as TopHat1 in the following) and Tophat2 [7] as these are the most commonly used RNA-seq mapping programs. Mapping results of these programs on the used data sets as well as evaluation scripts were provided by the authors of the RGASP study (https://github.com/RGASP-consortium/). For all programs, we evaluated the performance without and with an annotation (indicated by “ann”). For STAR, we evaluated both the 1- and 2-pass version. In the 2-pass version of STAR, splice junctions detected in the first run (1-pass) are taken as an input for a second run to improve mapping.

We applied the same evaluation scripts to evaluate ContextMap 2 mapping runs using Bowtie (version 0.12.7), Bowtie 2 (version 2.1.0), or BWA (version 0.7.8) as internal short read alignment programs. Additionally, we evaluated the performance of ContextMap 2 using BWA and an annotation. Here, the annotation is only used for scoring splice junctions when resolving overlapping splice sites (see Additional file 1: Supplementary methods). As for the RGASP evaluation, the annotation was taken from Ensembl version 62. Although we also performed evaluation of the original ContextMap implementation, we did not include it in the article as it performed worse in all evaluated metrics than ContextMap 2.

For runtime comparison, we applied all RNA-seq mapping programs with the same parameter settings as described in the RGASP study. The only exception was MapSplice. In this case, an internal version of MapSplice was used in the RGASP study, which is not available for download. Most likely it was an unfinished predecessor of MapSplice 2, which has since been made publicly available (http://www.netlab.uky.edu/p/bioinfo/MapSplice2). It was not the published MapSplice 1.x version as options were used (e.g. detection of indels with length >3) that this version does not support. We thus included an evaluation of MapSplice 2 in this article by applying it to all data sets using default parameters. Since MapSplice 2 uses the annotation only to detect fusion junctions between different genes, which was not simulated in the RGASP data sets, MapSplice 2 was only applied without annotation.

### Alignment yield

As a first metric, we evaluated the fraction of mapped reads for both simulated data sets (see Additional file 1: Table S2). This showed significant differences between RNA-seq mapping programs with GSNAP having the highest mapping rates (∼99% and 98% of the reads for simulation 1 and 2) and TopHat1/2 and ContextMap 2 having lowest mapping rates (89-96% of reads mapped in simulation 1 and 78-88% in simulation 2).

**Figure 4:**
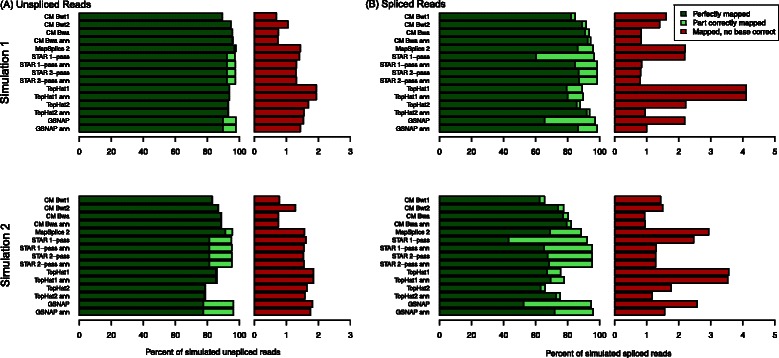
Fraction of perfectly mapped, part correctly mapped and incorrectly mapped reads for simulated unspliced **(A)** and spliced **(B)** reads of simulation 1 and 2, respectively. “CM Bwt1”, “CM Bwt2”, “CM Bwa” denote ContextMap 2 used with Bowtie, Bowtie 2, and BWA as underlying alignment program, respectively. If a gene annotation was provided, “ann” was added to the name of the respective program.

When investigating the fraction of reads mapped either perfectly, part correctly or with no base correct (Figure 4 and Additional file 1: Table S2), it became apparent that mapping rates alone are not meaningful for comparing the performance of algorithms. Despite GSNAP’s high overall mapping rate, the fraction of perfectly mapped reads was only 89% and 76% of reads of simulation 1 and 2, respectively. In contrast, ContextMap 2 using BWA mapped almost 95% and 87% of reads perfectly, which was better than for all other evaluated methods except MapSplice 2. Consistently, both the fraction of part correctly mapped reads and reads with no base mapped correctly were lower than for all other methods (see also Figure 4). Thus, the higher mapping rates of other programs came at the cost of higher error rates.

To investigate whether performance differed between unspliced and spliced reads, mapping rates were also calculated separately for both types of reads (Figure 4 and Additional file 1: Tables S3 and S4). Indeed, the evaluated programs differed considerably in performance between spliced and unspliced reads but not in any consistent fashion. For ContextMap 2 using Bowtie, MapSplice, STAR 1-pass, TopHat1 and GSNAP (and TopHat2 on simulation 1), the fraction of reads mapped completely wrong increased by more than 0.5 percentage points for spliced reads compared to unspliced reads. In contrast, this fraction did increase less for ContextMap 2 using Bowtie 2 or BWA (and TopHat2 on simulation 2) and even decreased for the remaining tools. In all cases, however, the number of part correctly mapped reads increased for spliced reads, but least for ContextMap 2 and TopHat2. This was likely due to a part of the read on one side of the splice junction not being mapped correctly or not at all (e.g. in case of STAR, which can also output clipped alignments). In particular for STAR and GSNAP, this lead to 10-50% part correctly mapped reads.

In summary, these results show that ContextMap 2 using BWA had the lowest rate of incorrectly mapped reads among all evaluated programs. Furthermore, it mapped more reads perfectly than any of the other programs except MapSplice 2. However, MapSplice 2 had ∼2-fold higher rates of incorrectly mapped reads.

Interestingly, we observed that the choice of the underlying alignment program had a significant influence on the performance in RNA-seq mapping. Both rates of perfectly and incorrectly mapped reads are improved significantly when using BWA within ContextMap 2 instead of either Bowtie or Bowtie 2. The reduced number of perfectly mapped reads for Bowtie is mostly due to its lower overall recall [14] and the fact that it does not determine local alignments and thus does not support the detection of multi-split read alignments and indels within ContextMap 2. The higher number of incorrectly mapped spliced reads results from spliced reads for which the seed at the read start cannot be aligned at the correct position, e.g. because the splice site in the read is closer to the read start than the seed length, but the seed can be aligned to a wrong position. In this case, no backward alignment is performed for the read in order to reduce runtime and only the incorrect alignments are further analyzed.

The lower mapping quality using Bowtie 2 compared to BWA resulted from the fact that – in contrast to Bowtie and BWA – Bowtie 2 has a dramatically increased runtime if the maximum number of valid alignments reported per read (-k option) is set to even moderately high values. Thus, per default we used a relatively low value of *k*=3. Using a value of *k*=10 resulted in comparable mapping quality to ContextMap 2 with BWA (see Additional file 1: Tables S2, S3 and S4) but runtime increased by at least 8 h compared to BWA or Bowtie 2 with *k*=3 (Table 1).

**Table 1:** **Runtime in CPU hours for each program on simulation 1 and 2**

**Program**	**Simulation 1**	**Simulation 2**
ContextMap Bwt1	11.67	11.02
ContextMap Bwt2 (*k*=3, default)	16.47	15.58
ContextMap Bwt2 (*k*=10)	24.98	24.55
ContextMap Bwa	11.58	14.00
ContextMap Bwa ann	11.92	14.15
MapSplice 2	31.43	28.62
STAR 1-pass	0.82	1.28
STAR 1-pass ann	1.05	1.58
STAR 2-pass	9.60	10.28
STAR 2-pass ann	9.57	10.80
TopHat1	20.1	28.43
TopHat1 ann	20.53	29.03
TopHat2	25.17	27.23
TopHat2 ann	34.32	39.68
GSNAP	147.73	128.15
GSNAP ann	160.78	140.27

All methods were run using 8 cores on the same machines and with the same parameter settings as in the RGASP evaluation [17]. ContextMap 2 with Bowtie 2 was run with the maximum number of alignments reported per read (*k*) set to 3 (default setting used for evaluating mapping quality) and 10, respectively. Runtime of STAR 2-pass includes the time required for running STAR 1-pass, indexing the genome with splice sites found in the first STAR run and re-running STAR.

### Alignment yield on real-life RNA-seq data

**Figure 5:**
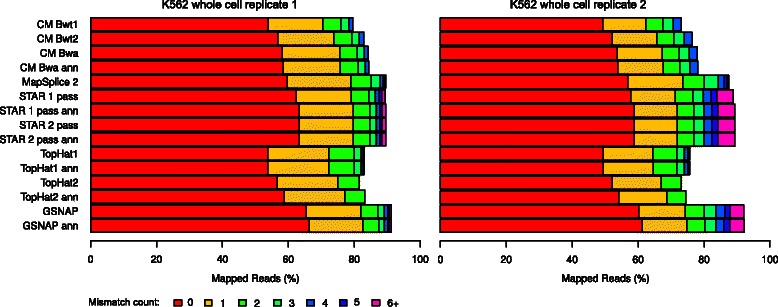
Percentage of mapped reads and mismatch distribution for the mapped reads for both replicates of the K562 whole cell RNA-seq samples. Results for all real-life samples are shown in Additional file 1: Figure S4.

Consistent with evaluation results on simulated data, alignment yield of ContextMap 2 was lower on all samples for the K562 cell line than for MapSplice 2, STAR or GSNAP, but similar or slightly higher than for TopHat1/2 (Figure 5 and Additional file 1: Figure S4). This was only partly due to the relatively small number of mismatches (= 4) allowed per default in ContextMap 2.

Nevertheless, the ranking of algorithms with regard to the number of mapped reads is quite similar to the ranking on the simulated data. Thus, if we also extrapolate the results on perfectly and incorrectly mapped reads from the simulation to the real-life data, this would suggest that the difference in mapped reads between ContextMap 2 and most other mapping programs are to a large extent due to incorrect mappings identified by the other programs.

### Spliced alignment

**Figure 6:**
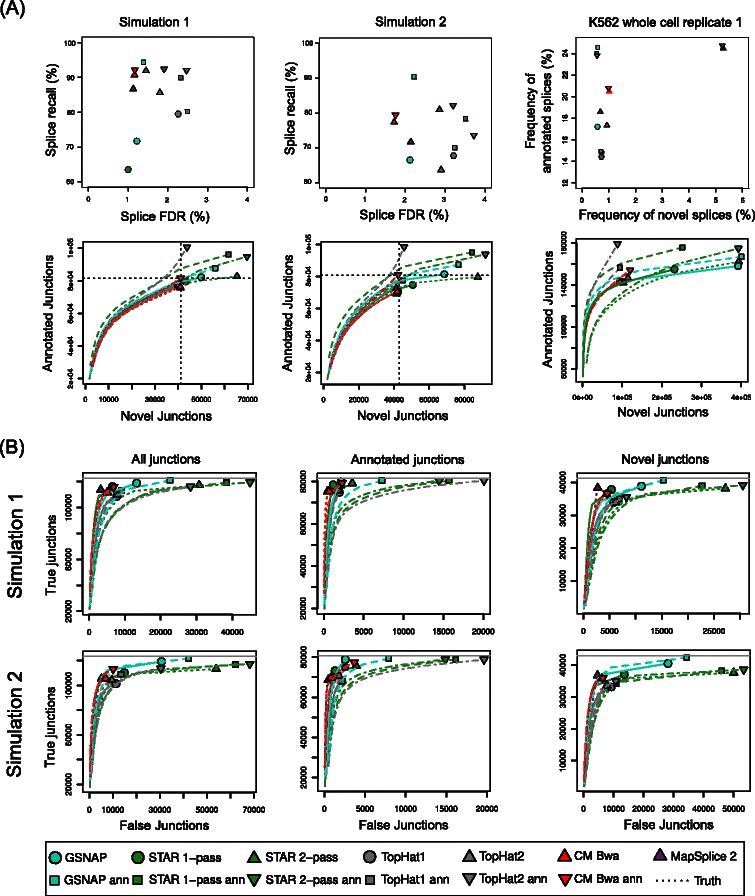
Evaluation of splice junction prediction.**(A)** Comparison of splice recall (y-axis) versus splice false discovery rate (FDR=1-precision, x-axis) on simulation 1 and 2 (see equations 2 and 3 for definitions). For the human data sets, the frequency of predicted novel splices was compared to the frequency of annotated splices for the Ensembl annotation (see text for definitions, Additional file 1: Figure S5 for results for all real-life data sets). Furthermore, the number of identified annotated and novel junctions was evaluated (see Additional file 1: Figure S6 for results for all data sets). To obtain receiver operation characteristic (ROC)-like curves, numbers were also calculated at increasing thresholds on the number of supporting reads for each junction. **(B)** Number of correctly predicted (true) and incorrectly (false) junctions were compared for all junctions and annotated and novel junctions separately. In contrast to the RGASP evaluation, we also included junctions covered by only 1 read. ROC-like curves were calculated as in **A**. **(A-B)** For ContextMap 2 only results using BWA are shown, results for Bowtie and Bowtie 2 can be found in Additional file 1: Figures S5 and S6 (for **A**) and S7 (for **B**).

Since performance on spliced reads showed the largest differences among the mapping approaches, these were analyzed in more detail (Figure 6A and Additional file 1: Figure S5). For this purpose, splice recall and false discovery rate (FDR) were calculated as in the original RGASP study. Here, splice recall is defined as

(2) $$ \begin{aligned} \text{recall}&=\frac{\# \textrm{true positive splices}}{\# \textrm{simulated splices}} \\ &=\frac{\# \textrm{true positive splices}}{\# \textrm{true pos. splices} + \# \textrm{false neg. splices}}. \end{aligned}  $$

In this case, a splice is defined as one junction in one particular read. Thus, if a simulated junction within a read is recovered by the alignment for this read, it is considered a true positive splice. If it is not recovered, it is a false negative splice. If the alignment contains a junction that was not simulated for this read, it is considered a false positive splice. FDR is then defined as 1 - precision, with

(3) $$ \begin{aligned} \text{precision}&=\frac{\# \textrm{true positive splices}}{\# \textrm{predicted splices}} \\ &=\frac{\# \textrm{true positive splices}}{\# \textrm{true pos. splices} + \# \textrm{false pos. splices}}. \end{aligned}  $$

For the real data, recall and FDR could not be calculated as the correct mapping was not known. Instead, the fraction of reads mapping to an annotated splice junction (=: frequency of annotated splices) was compared to the fraction of reads mapping to a novel splice junction (=: frequency of novel splices).

Consistent with the evaluation of alignment yield, this analysis showed that ContextMap 2 combined low FDR with high recall. Again the combination with BWA performed best. Although some of the other mapping programs showed higher recall, this was always accompanied by significantly higher FDR. Generally, the increase in recall compared to ContextMap 2 was only modest with the exception of annotation-based GSNAP on simulation 2.

The analysis of known and novel splices identified in the real data set showed that ContextMap 2 mapped reads to novel splices with similar frequency as most other programs except STAR 2-pass (Figure 6A and Additional file 1: Figure S5). In contrast, reads were mapped to known splice junctions less frequently compared to most programs using an annotation and more frequently than most programs without annotation. Unfortunately, these results are difficult to interpret as alignments to novel junctions are not necessarily wrong and alignments to annotated junctions not necessarily right.

To address this problem we also compared the number of novel and annotated junctions predicted by all methods between the simulations and the real data sets (Figure 6A and Additional file 1: Figure S6). Here, the same junction (in terms of the genomic coordinates) identified for several reads was counted only once. This consistently showed that ContextMap 2 predicted significantly fewer novel junctions than STAR and GSNAP (>50% less). Here, ContextMap 2 using BWA or Bowtie 2 and MapSplice showed quite similar performance, whereas annotation-based ContextMap 2 using BWA and, in particular, annotation-based TopHat2 predicted significantly more annotated junctions. Interestingly, annotation-based ContextMap 2 identified almost precisely the correct number of annotated and novel junctions for both simulations. The high similarity of the results between simulation and real data indicates that recall and FDR from the simulations can again be extrapolated to the real data sets. This would suggest that ContextMap 2 using BWA (both with and without an annotation) correctly identifies more reads with known junctions than programs not using an annotation but is less biased towards annotated junctions than other programs using an annotation.

This conclusion is also supported by the comparison of the number of correctly predicted junctions to false junctions (Figure 6B and Additional file 1: Figure S7). This again shows that ContextMap 2 (in particular when using BWA) predicts much fewer false junctions than approaches using an annotation, while missing relatively few of the true junctions. For novel junctions ContextMap 2 is only outperformed in terms of recall and FDR by MapSplice 2, but the difference in performance is relatively small. For annotated junctions, the ContextMap 2 version without annotation performs almost as good as MapSplice 2, which has the lowest FDR, whereas the version using the annotation has a significantly higher recall but also predicts more false junctions. Again, this highlights the problem in using an annotation, which might bias the results towards known junctions. Nevertheless, ContextMap 2 appears to be less biased by the annotation than STAR, GSNAP or Tophat2.

### Detection of multi-junction reads

Since ContextMap 2 now also supports mapping of reads crossing multiple junctions, we calculated recall and precision separately for reads containing different number of junctions (Table 2 and Additional file 1: Table S5). For this purpose, a read was considered a true positive if all junctions in this read were identified correctly and no additional junctions were predicted. If a different number of junctions were predicted than correct, it was considered a false negative for this junction number and a false positive for the junction number predicted by the alignment. If the correct number of junctions were predicted for the read, but some of the junctions were wrong, it was considered a false positive for this junction number. To evaluate the trade-off between precision and recall, we calculated F-measure values defined as

(4) $$ F-measure= 2\cdot \frac{precision \cdot recall}{precision +recall}.  $$

**Table 2:** **F-measure [in %] for spliced reads with different number of spanned junctions for simulation 1**

**Program**	**Number of junctions spanned**				
	**1 (13808336)**	**2 (598297)**	**3 (11781)**	2* (548382)	3* (6908)
CM Bwt1	91.47	14.24	-	15.16	-
CM Bwt2	94.03	78.47	50.21	82.37	72.66
CM Bwa	95.03	82.73	53.33	86.67	76.46
CM Bwa ann	95.74	84.65	53.9	88.47	76.79
MapSplice 2	92.42	79.18	27.27	80.65	3.44
STAR 1-pass	77.63	30.91	5.01	31.91	1.49
STAR 1-pass ann	93.55	81.65	75.71	82.57	82.17
STAR 2-pass	95.0	85.55	82.07	86.59	87.29
STAR 2-pass ann	95.07	86.28	82.55	87.02	86.49
TopHat1	87.83	77.51	63.57	80.42	75.56
TopHat1 ann	88.02	78.99	68.13	81.06	76.1
TopHat2	91.71	87.0	76.92	89.66	88.51
TopHat2 ann	94.84	90.79	85.92	92.05	90.35
GSNAP	83.13	43.45	18.52	42.35	12.29
GSNAP ann	96.47	88.59	79.51	89.86	84.67

^*^The last two columns show results only for reads for which all exons except the first and last exon had length ≥ 20 nt. For this evaluation, read alignments were only considered a true positive if all simulated splice junctions in the read were recovered and no additional splice junctions were identified. Indels were ignored for this purpose. Recall and precision values for both simulations can be found in Additional file 1: Table S5.

This showed that ContextMap 2 using BWA (both with and without annotation) outperformed all other programs on reads containing only one junction except STAR 2-pass and annotation-based GSNAP. This includes the vast majority of all spliced reads. Here, only annotation-based GSNAP performed significantly better, at least on simulation 2. In general, F-measure decreased with increasing number of junctions for all programs, mostly due to lower recall values. Precision generally remained above 90%. For reads with two junctions, ContextMap 2 with BWA was still only outperformed by STAR 2-pass, annotation-based GSNAP and now also annotation-based Tophat2, but the difference in recall to these programs increased.

For three junctions, however, recall and thus F-measure of ContextMap 2 using BWA or Bowtie 2 dropped dramatically, such that only MapSplice 2, STAR 1-pass and GSNAP (both without annotation) performed worse. Since Bowtie does not perform local alignment, ContextMap using Bowtie cannot identify multi-split alignments and therefore had zero recall on three-junction reads. A small number of two-junction reads were mapped as single-split alignments are extended to multi-split alignments in step 3 of ContextMap 2 if they overlap an additional splice site.

Since ContextMap 2 by default only determines multi-split alignments for which internal exons are at least 20 nt long (= minimum exon size *e*), we repeated the analysis only for multi-junction reads fulfilling this condition. The results of this analysis are shown in the last two columns of Table 2 and Additional file 1: Table S5. Here, ContextMap 2 using BWA showed a significant improvement, resulting in similar or better performance for two-junction reads than all programs except TopHat2 on simulation 1 and annotation-based GSNAP. For three-junction reads, recall of ContextMap 2 was almost doubled, whereas for other programs improvements were less pronounced and recall of MapSplice 2 actually decreased to <2%. In addition, ContextMap 2 using BWA generally showed a significantly higher precision than the programs with particularly high recall.

### Indel accuracy

**Figure 7:**
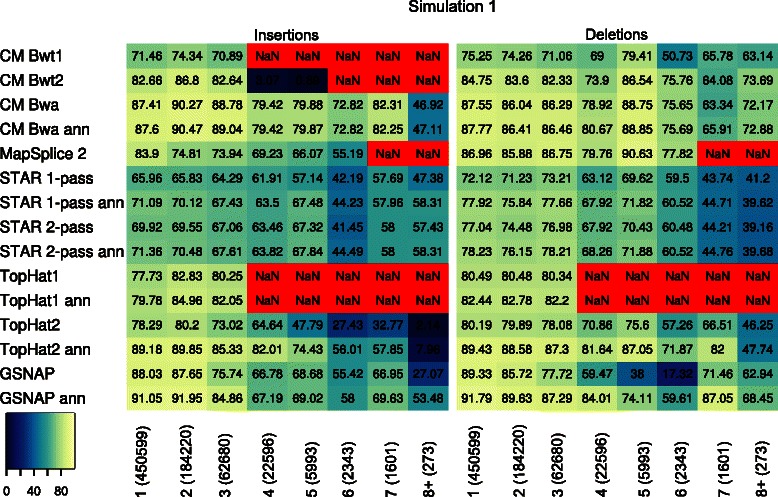
F-Measure [in %] for insertion and deletions identified by all programs on simulation 1. NaN indicates that no insertion or deletion of that size was identified. Insertion and deletion size are shown below each column of the heatmap. The numbers in parentheses indicate the number of simulated reads for each insertion or deletion size. Results for simulation 2 are shown in Additional file 1: Figure S8. Recall and precision values are listed in Additional file 1: Tables S6 and S7.

Precision, recall and F-measure values were also calculated separately for reads containing insertions and deletions (Figure 7, Additional file 1: Figure S8, and Additional file 1: Tables S6 and S7). These results show that ContextMap 2 using BWA outperforms all other approaches on both insertions and deletions except for GSNAP (both with and without annotation) and annotation-based TopHat2. Furthermore, the latter programs only performed comparably well to ContextMap 2 on reads with small indel size (1-4, depending on the method). In almost all cases, precision of ContextMap 2 using BWA was above 90% and higher than for the best competing programs. Similar to multi-junction reads, the integration of Bowtie or Bowtie 2 in ContextMap 2 resulted in worse performance on indels than for BWA, in particular for longer insertions.

**Figure 8:**
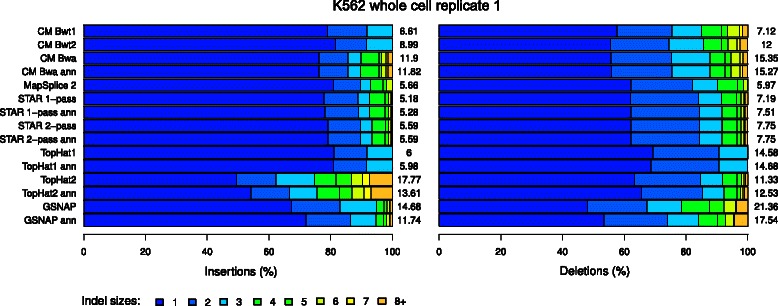
Fraction of mapped reads with different indel sizes among all reads with indels for the first replicate of the K562 whole cell sample. Numbers next to the barplots indicate the number of mapped reads with indels divided by 10^5^ (i.e. number of reads per 100,000). Results for all samples are shown in Additional file 1: Figures S9 and S10.

Numbers of detected indels and indel length were also evaluated on the real-life sequencing data (Figure 8 and Additional file 1: Figures S9 and S10). Consistent with their higher recall on the simulations, ContextMap 2 using BWA, TopHat2 and GSNAP mapped at least twice as many reads with insertions than the other programs. Interestingly, numbers of mapped insertions generally decreased significantly for TopHat2 and GSNAP when not using an annotation, while there were hardly changed for ContextMap 2 using BWA. Since simulation results showed higher precision for annotation-based GSNAP and TopHat2 compared to the runs without annotation but not lower recall, this indicates that the lost mappings were largely false positive results. Furthermore, even compared to annotation-based GSNAP and TopHat2, precision of ContextMap 2 was higher on the simulations (in particular for long insertions, which were enriched among TopHat2 results) indicating that many of the insertions additionally identified by these competing tools were not correct.

With regard to deletions, only GSNAP consistently recovered more reads with deletions than ContextMap 2 using BWA and again numbers decreased for annotation-based GSNAP. As the latter had both higher recall and precision on the simulations than GSNAP alone, this again suggests that the difference in mapped reads between GSNAP with and without annotation were false positives. Compared to ContextMap 2, annotation-based GSNAP identified a higher fraction of longer deletions. As the simulations showed a significantly lower precision, in particular on long deletions, for GSNAP, this again indicates that a significant fraction of the additional reads with deletions identified by annotation-based GSNAP are incorrectly mapped.

### Runtime comparison

Finally, we compared runtime between all evaluated programs on the simulated data sets (Table 1). Here, ContextMap 2 was much faster than all evaluated programs except STAR 1- and 2-pass. Here, STAR 1-pass was extremely fast, whereas STAR 2-pass was only ∼20-24% faster than ContextMap 2. However, the evaluation on the RGASP data showed that this improved runtime came at the cost of both lower precision and recall for all STAR variants, in particular STAR 1-pass, compared to ContextMap 2.

Highest runtime of all evaluated approaches was observed for GSNAP with >128 CPU hours, i.e. more than 5 days. Thus, although it performed well on the detection of multi-junction reads and indels, runtime is too large for practical purposes. Among the remaining competing approaches, MapSplice 2 performed best in the evaluation of alignment quality, but not consistently better than ContextMap 2 using BWA. With regard to runtime, however, it performed significantly worse with ∼30 CPU hours on both simulations compared to 11-16 CPU hours used by ContextMap 2. Here, lowest runtime was observed when using Bowtie and highest using Bowtie 2, in particular when increasing the maximum number of reported alignments *k* to 10. Thus, BWA is the best choice as integral alignment algorithm for ContextMap 2 taking into account mapping quality and runtime.

## Conclusion

In this article, we presented ContextMap 2, a new and improved version of the context-based RNA-seq mapping program ContextMap. The key novel features of ContextMap 2 are the plug-in structure, which allows integrating new developments in short read alignment, as well as the detection of multi-split alignments, insertions and deletions. Performance of ContextMap 2 integrating either Bowtie, Bowtie 2 or BWA was evaluated on data sets from the recent RGASP evaluation of RNA-seq mapping programs and compared to the best performers of this study.

This showed that performance of RNA-seq mapping can be improved substantially by replacing the internal short read alignment program by more recent methods or versions. In this case, the use of BWA as integral alignment program generally improved recall and precision of ContextMap 2 compared to Bowtie and Bowtie 2 at only slightly higher or even lower runtime, respectively. Here, the plug-in structure of ContextMap 2 allows the extension to future versions of these alignment programs or even newly developed short read alignment programs with improved accuracy or runtime. Furthermore, this extension can also be performed by developers of such programs or other users of ContextMap 2 by simply implementing the interface. In contrast, other existing RNA-seq alignment programs are limited to one or at most two short read alignment programs. For instance, MapSplice 2 still uses only Bowtie and TopHat2 only supports Bowtie and Bowtie 2.

ContextMap 2 with BWA performed similarly well or better than other state-of-the-art RNA-seq mapping programs with regard to perfectly mapped reads on simulated data, while having at least ∼2-fold lower rates of reads mapped only part correctly or at completely wrong positions. Thus, reduced mapping rates of ContexMap 2 on both simulated and real data can be mostly explained by lower rates of incorrectly mapped reads. ContextMap 2 using BWA showed high precision and recall on all evaluated tasks, in particular on the detection of long insertions and deletions. Furthermore, runtime was generally at least 50% lower than for the best competing programs. Only STAR 1- and 2-pass were faster, but showed significantly lower precision, in particular on spliced reads and splice junctions, and low recall on reads containing indels.

## Availability and requirements

**Project name:** ContextMap 2 **Project home page:** http://www.bio.ifi.lmu.de/ContextMap **Operating system(s):** Platform independent **Programming language:** Java **Other requirements:** Java 7 or higher; one of the following: Bowtie version 0.12.7 or higher, Bowtie 2 version 2.1.0 or higher, BWA version 0.7.8 or higher **License:** Artistic License **Any restrictions to use by non-academics:** none

## Additional file

Additional file 1 **Supplementary material.** Supplementary material contains Supplementary methods, Figures and Tables.
