# Supplementary material for: ContextMap 2: fast and accurate context-based RNA-seq mapping
Source: BMC Bioinformatics. 2015 Apr 17;16:122. doi: 10.1186/s12859-015-0557-5 (PMC4411664; doi:10.1186/s12859-015-0557-5)
Supplement: Supplementary file 1 — Supplementary material. Supplementary material contains Supplementary methods, Figures and Tables. [file 12859_2015_557_MOESM1_ESM.pdf]

SUPPLEMENTARY MATERIAL

ContextMap 2: Fast and accurate context-based RNA-seq mapping

Supplementary Methods and Figures

Alignment extension for split alignments

In step 3 (Figure 1 C in the main manuscript) of ContextMap 2, additional alignments are created within each context based on the initial alignments determined in step 1. For partial and full alignments, this is described in the main manuscript. Similarly, additional alignments are determined for split alignments. The input for this extension is the set of single-split alignments determined in step 1. Each single-split alignment consists of a combination of continuous alignments beginning at the read start and the read end separated by the predicted intron (Supplementary Figure 1 A). The ends of this intron represent the predicted splice sites. Here, step 1 determines only one split alignment with the minimum number of mismatches for each combination of alignment start (anchor A in Supplementary Figure 1 A) and alignment end (anchor B in Supplementary Figure 1 A).

However, other split alignments may be possible for the same combination of alignment start and end with the same number of mismatches or only a few more (the difference in mismatches allowed is provided by the user using the maximum mismatch difference [*mmdiff*] parameter). These alignments are determined in step 3 of ContextMap 2 by shifting the position of the splice sites as shown in Supplementary Figure 1 B.

Furthermore, single-split alignments are checked for overlaps with split alignments of other reads indicating an additional split within the continuously aligned regions. If an overlap is found, the single-split alignment is extended to a multi-split alignment if the number of mismatches does not exceed the predefined maximum mismatch criterion. Here, only single-split alignments of the whole read are extended to two-split alignments, but not single-split alignments of read fragments obtained for candidate multi-split alignments. As a consequence, ContextMap 2 can determine multi-split alignments with two junctions for which the internal exon is shorter than the minimum exon size e used for defining the fragments in the detection of multi-split alignments. Multi-split alignments with more than two splits and at least one internal exon shorter than e are not found.

This results in a set of alternative split alignments for each read with at most the maximum number of allowed mismatches. Resolution of these multiple alignments is then performed in step 4 and 5 of ContextMap 2.

Resolution of overlapping splice sites

This is part of step 4 of ContextMap 2, in which multiple alignments are resolved within contexts. Here, splice sites are eliminated which are very close to each other and suggested by alternative split alignments of the same read with the same alignment start and end but different position of the splice site. Elimination is based on the evidence for different splice sites provided by all reads. Although it might appear counterintuitive that alternative split alignments are first created in step 3 of ContextMap 2 and then some are deleted again, this guarantees that all reads with a valid split alignment using this splice site are included in calculating the evidence score.

Two splice sites $(s_{1,1}, s_{1,2})$ and $(s_{2,1}, s_{2,2})$ are considered overlapping if both $|s_{1,1} - s_{2,1}|$ and $|s_{1,2} - s_{2,2}|$ are smaller than the maximum read length. Here, $s_{i,1}$ denotes the genome position of the end of the first exon and $s_{i,2}$ the start of the second exon. While in the original ContextMap implementation only one splice site from a set of overlapping splice sites was used, ContextMap 2 retains the three splice sites

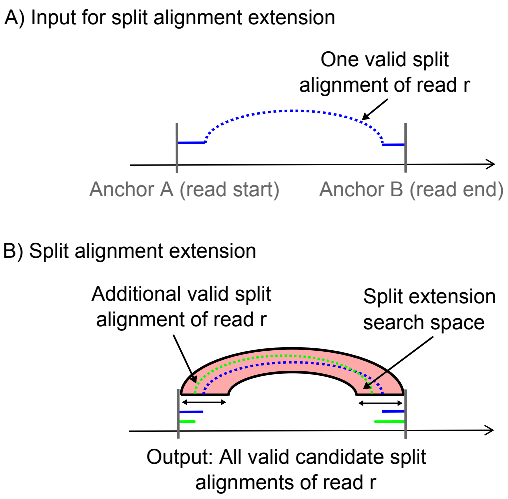

Supplementary Figure 1 (A) The single-split detection method in step 1 provides only one valid split alignment with the minimum number of mismatches for each possible combination of alignment start and end. (B) By shifting the position of the splice site, alternative split alignments with the same number of mismatches or a few more (indicated by the maximum mismatch difference parameter) are determined. The range of the search space is defined by the read length.

from each set with the highest evidence score (see Supplementary Figure 2). This allows the detection of alternative 3' or 5' splice sites.

ContextMap 2 uses a similar evidence score as in the original ContextMap version. The major difference involves the treatment of gene annotation (if provided) and known splice signals. If at least one splice site within the set of overlapping splice sites corresponds to an annotated exon-exon junction or shows a known splice signal, all other splice sites not corresponding to a known exon-exon junction or having no known splice signal are discarded. The evidence score used for evaluating the remaining splice sites is calculated as follows. Let n_i be the number of reads (full, split or partial) with i mismatches supporting the splice site pair and m the maximum number of mismatches allowed. Then the evidence score is defined as:

$$evidence = \sum_{i=0}^{m} (w^i \cdot n_i) \qquad (1)$$

Here, w is a value < 1 (default $w = 0.3$). Thus, the score is the weighted sum of the number of reads with the weight decreasing exponentially with the number of mismatches.

For each set of pairwise overlapping splice sites, the three splice sites with the highest evidence scores are selected. Split read alignments containing the discarded splice sites are discarded. If more than one split alignment remains for a read to any of the remaining three splice sites, the split alignment to the splice site with highest evidence score is retained and all others discarded.

Resolution of multiple read alignments

ContextMap 2 resolves multiple read alignments first within each context in step 4 and subsequently between the contexts in step 5. For this purpose, a support score is calculated for each alignment based on the number of reads aligned within and around the context. In the original ContextMap implementation,

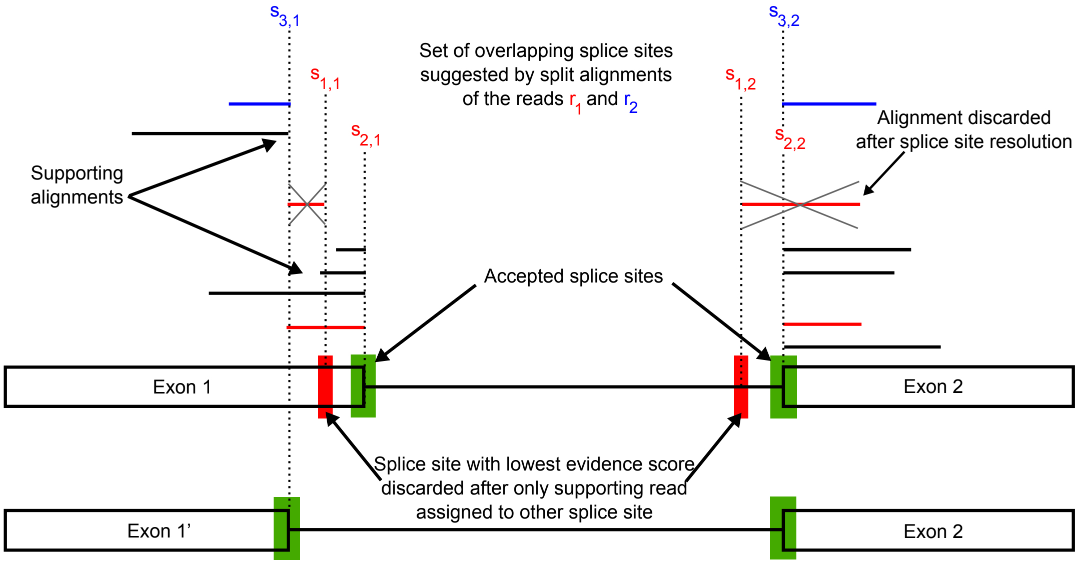

Supplementary Figure 2 Resolution of overlapping splice sites. Split alignments of reads r_1 (red) and r_2 (blue) suggest a set of three overlapping splice sites $\{(s_{1,1}, s_{1,2}), (s_{2,1}, s_{2,2}), (s_{3,1}, s_{3,2})\}$. Here, $(s_{1,1}, s_{1,2})$ and $(s_{2,1}, s_{2,2})$ are indicated by alternative split alignments of the same read r_1. Assuming that all shown alignments have zero mismatches, this results in the following evidence scores for the three splice sites: 1, 4 and 2. Although all three splice sites would be retained at first, the only supporting read for the splice site $(s_{1,1}, s_{1,2})$, i.e. r_1, is assigned to the splice site $(s_{2,1}, s_{2,2})$ with higher evidence score. As a consequence, the splice site $(s_{1,1}, s_{1,2})$ is discarded as it is no longer supported by any reads.

the score was defined as (see Supplementary Figure 3 A)

$$support = \sum_{i=1}^{4} 2^{4-i} \cdot \lfloor \ln(score_i) \rfloor. \tag{2}$$

Here, $score_1$ was defined as the maximum number of reads mapping to any position within the region the read is aligned. $score_2$ was the maximum in a window of 200 nt either upstream of the read start or downstream of the read end. $score_3$ was the maximum > 200 but ≤ 500 nt from read start or end. Finally, $score_4$ was the maximum > 500 but ≤ 1000 nt from read start or end.

To better distinguish between different alignments for a read that are identical on one but not the other side of the read, the support score in ContextMap 2 was modified such that maximum read counts on both sides of the of read alignment are included separately in the score. Furthermore, we reduced the number of considered windows around the read alignment and their respective sizes since the considered region was much larger than an average exon (Supplementary Figure 3 B). The new support score is then defined as

$$\begin{aligned} support &= 2^3 \cdot \lfloor \ln(score_1) \rfloor \\ &+ \sum_{i=2}^{3} 2^{4-i} \cdot \left(\lfloor \ln(score_{-i}) \rfloor + \lfloor \ln(score_i) \rfloor \right). \end{aligned} \tag{3}$$

Here, $score_{-i}$ and $score_i$ were defined as the maximum read counts in the corresponding intervals upstream and downstream of the read alignment, respectively. For reads with multiple alignments, the

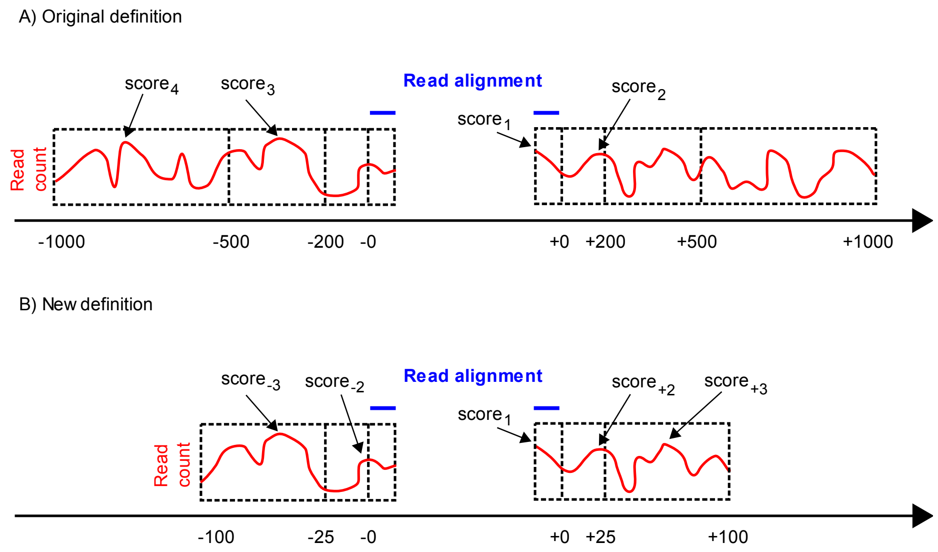

Supplementary Figure 3 Illustration of the old (A) and new (B) support score definition. The definition for full alignments is the same, with the exception that the region to which the read is aligned is continuous.

count was 1/(#multiple alignments of read within context) for step 4 and 1/(#multiple alignments of read between contexts) for step 5 of ContextMap 2.

Thus, if many reads are aligned to the same region, indicating that this region is actually expressed, the score of the alignment is high. If only few other reads are aligned to the same region as the read, the score of the alignment is low. Among several alternative alignments for the same read within each context, the one with the largest support score is then chosen. Finally, reads aligned to several different contexts are resolved in the same way in step 5 after recalculating support scores based on the read alignments chosen for each context.

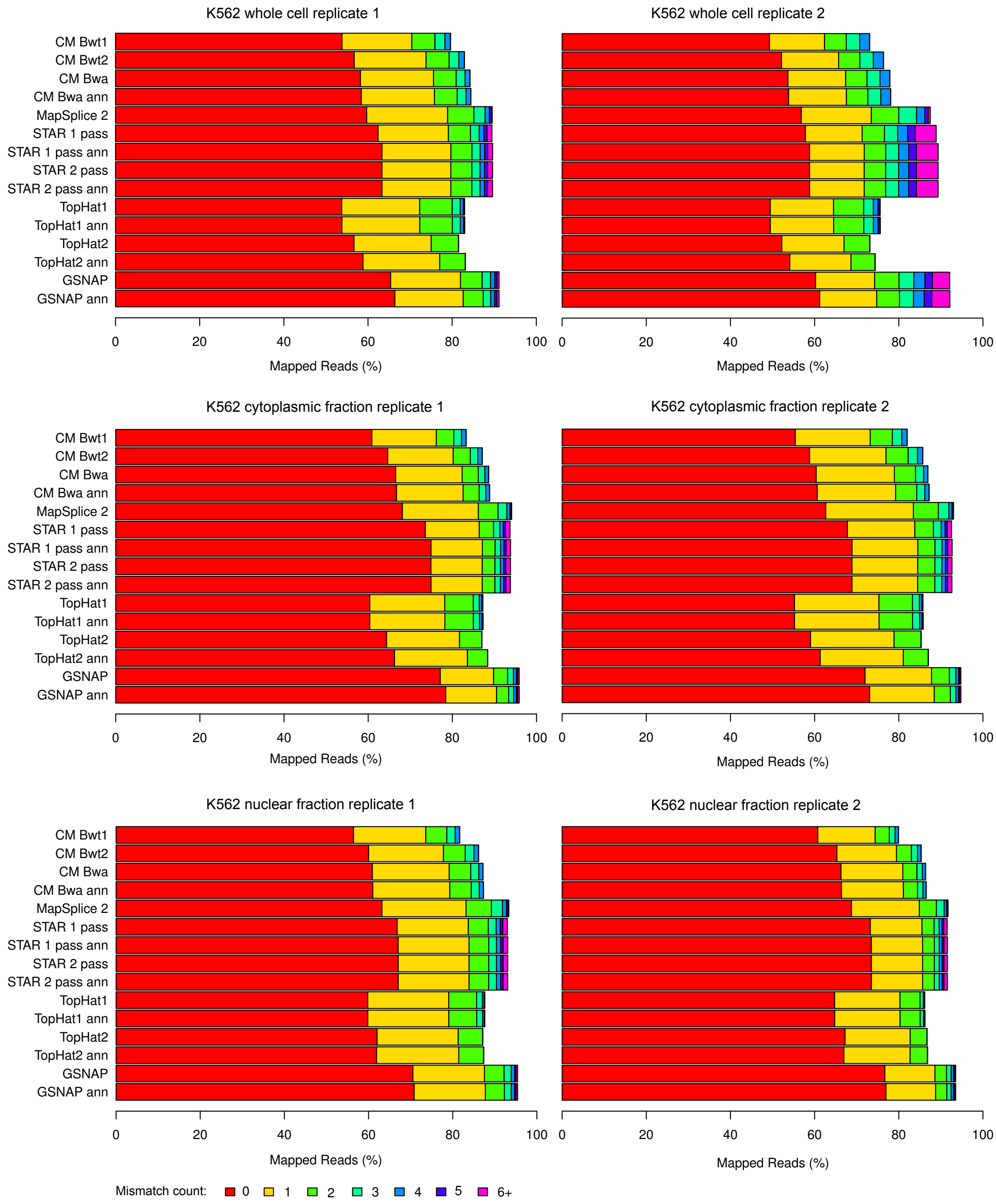

Supplementary Figure 4 Percentage of mapped reads and mismatch distribution for the mapped reads for all evaluated real-life data sets.

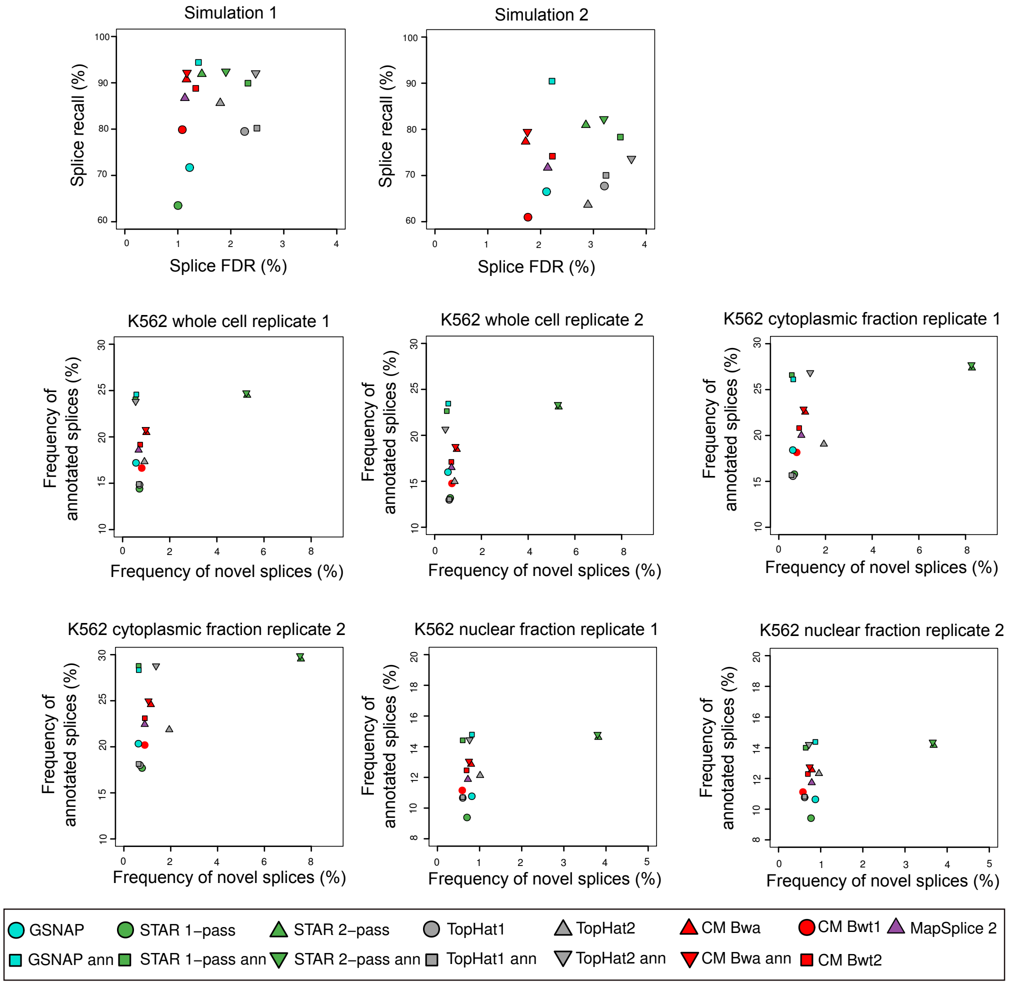

Supplementary Figure 5 Top row: Comparison of splice recall (y-axis) versus splice false discovery rate (FDR=1-precision, x-axis) on simulation 1 and 2 for all evaluated RNA-seq mapping programs. Bottom rows: Comparison of the frequency of predicted novel splices to the frequency of annotated splices for the Ensembl annotation for all evaluated real-life data sets and all evaluated RNA-seq mapping programs. See main manuscript for definitions.

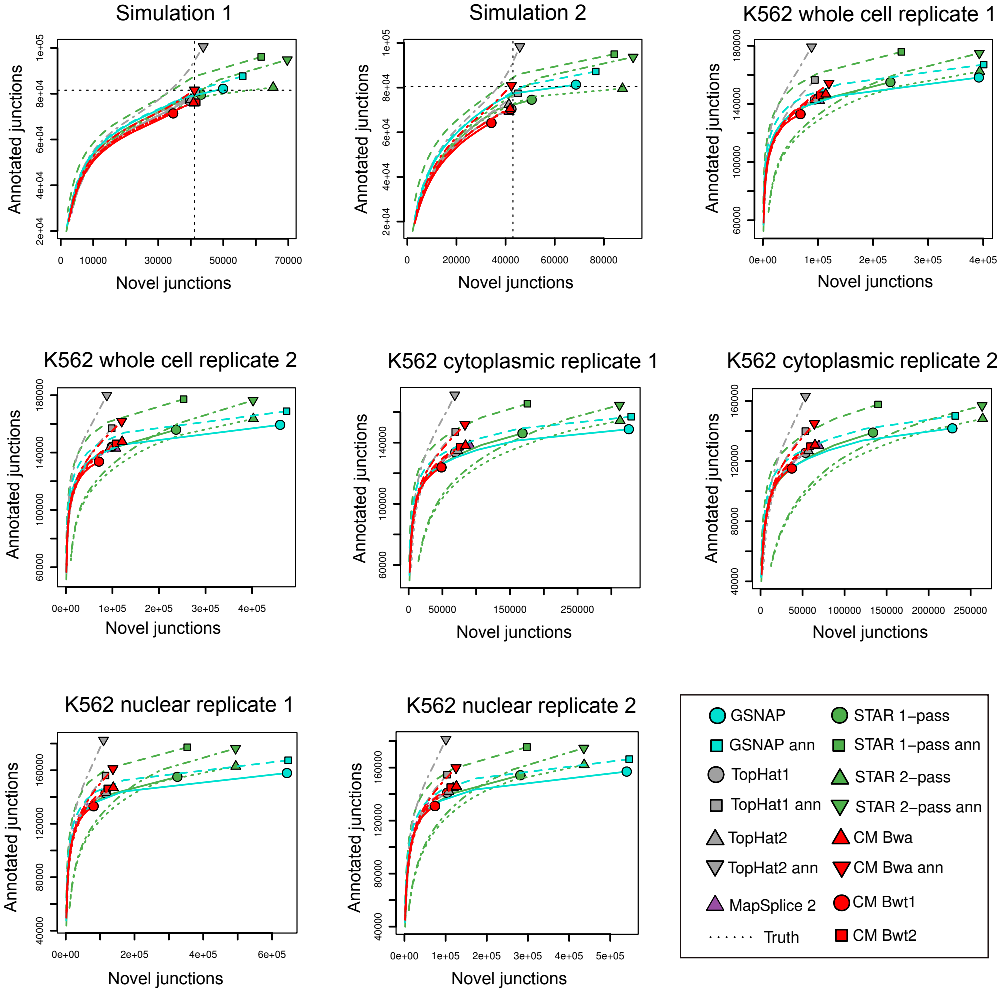

Supplementary Figure 6 Comparison of the number of annotated and novel junctions for all evaluated data sets and all evaluated RNA-seq mapping programs. To obtain receiver operation characteristic (ROC)-like curves, numbers were also calculated at increasing thresholds on the number of supporting reads for each junction.

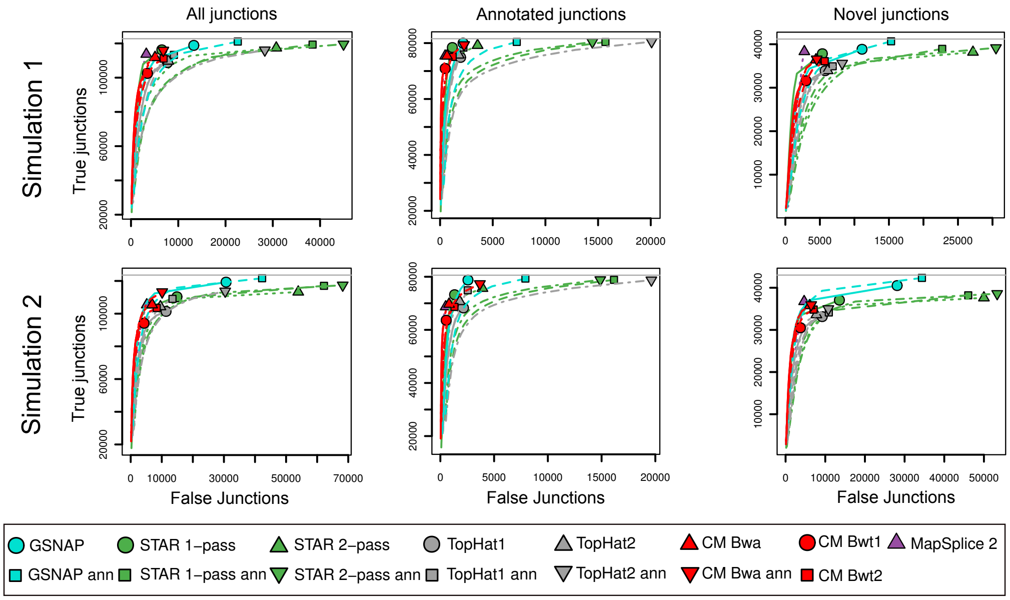

Supplementary Figure 7 Comparison of true and false junctions for all evaluated RNA-seq mapping programs. Number of correctly predicted (true) and incorrectly (false) junctions were compared for all junctions and annotated and novel junctions separately (symbols). To obtain receiver operation characteristic (ROC)-like curves, numbers were also calculated at increasing thresholds on the number of supporting reads for each junction. In contrast to the RGASP evaluation, we also included junctions covered by only 1 read.

Simulation 2

	Insertions								Deletions							
	1 (2847995)	2 (1011935)	3 (382986)	4 (144059)	5 (92747)	6 (13470)	7 (5748)	8+ (1853)	1 (2847995)	2 (1011935)	3 (382986)	4 (144059)	5 (92747)	6 (13470)	7 (5748)	8+ (1853)
CM Bwt1	58.12	58.39	57.87	NaN	NaN	NaN	NaN	NaN	62.58	61.34	63.21	62.48	62.53	61.69	73.87	55.68
CM Bwt2	72.58	72.45	73.05	0.15	0.16	NaN	NaN	0.43	74.56	74.56	73.73	74.95	75.36	73.69	80.19	66.68
CM Bwa	77.85	78.28	78.36	74.36	75.44	56.72	73.74	62.53	78.97	78.12	78.42	77.27	77.03	78.33	83.82	62.15
CM Bwa ann	78.05	78.42	78.47	74.44	79.48	57.24	73.78	63.64	79.13	78.32	78.58	77.37	77.28	78.55	84.1	60.99
MapSplice 2	69.46	59.28	59.15	54.48	59.04	47.77	NaN	NaN	72.92	72.9	72.87	72.55	68.94	54.56	NaN	NaN
STAR 1-pass	55.77	51.85	51.11	47.2	53.42	36.97	49.78	30.33	61.22	60.25	59.72	58.97	53.27	49.87	51.44	46.82
STAR 1-pass ann	61.12	56.55	55.18	50.22	54.11	37.89	54.7	32.62	65.82	65.2	63.42	61.84	57.85	56.09	53.47	42.85
STAR 2-pass	60.55	56.41	55.08	49.83	54.14	41.09	54.3	32.25	65.16	64.56	63.14	61.48	55.78	54.76	51.51	48.22
STAR 2-pass ann	61.64	57.12	55.82	50.55	54.15	38.05	55.06	32.51	66.09	65.6	63.71	62.14	57.75	56.38	52.82	44.77
TopHat1	66.83	69.35	68.41	NaN	NaN	NaN	NaN	NaN	72.05	71.66	70.8	NaN	NaN	NaN	NaN	NaN
TopHat1 ann	68.95	71.52	70.26	NaN	NaN	NaN	NaN	NaN	73.76	73.7	72.62	NaN	NaN	NaN	NaN	NaN
TopHat2	69.3	68.56	67.19	61.29	69.79	45.3	38.29	11.11	70.21	69.08	66.36	64.16	58.29	59.2	62.96	52.38
TopHat2 ann	79.08	79.02	78.6	78.29	77.83	58.51	57.14	13.42	78.12	77.43	75.48	73.65	68.9	71.82	70.1	49.55
GSNAP	85.08	85.23	83	57.69	64.26	55.15	60.54	43.57	85.69	85.01	81.95	76.79	68.11	51.55	71.65	53.13
GSNAP ann	86.44	87.04	85.95	58	64.45	55.95	62.32	47.32	86.85	86.5	84.41	83	79.21	71.99	79.12	58.66

0 40 80

Supplementary Figure 8 F-Measure [in %] for insertion and deletion prediction for all programs on simulation 2. NaN indicates that no insertion or deletion of that size were predicted. Insertion and deletion size are shown below the column of the heatmap. The numbers in parentheses indicate the number of simulated reads for each insertion or deletion size. Recall and precision values are listed in Supplementary Tables 6 and 7.

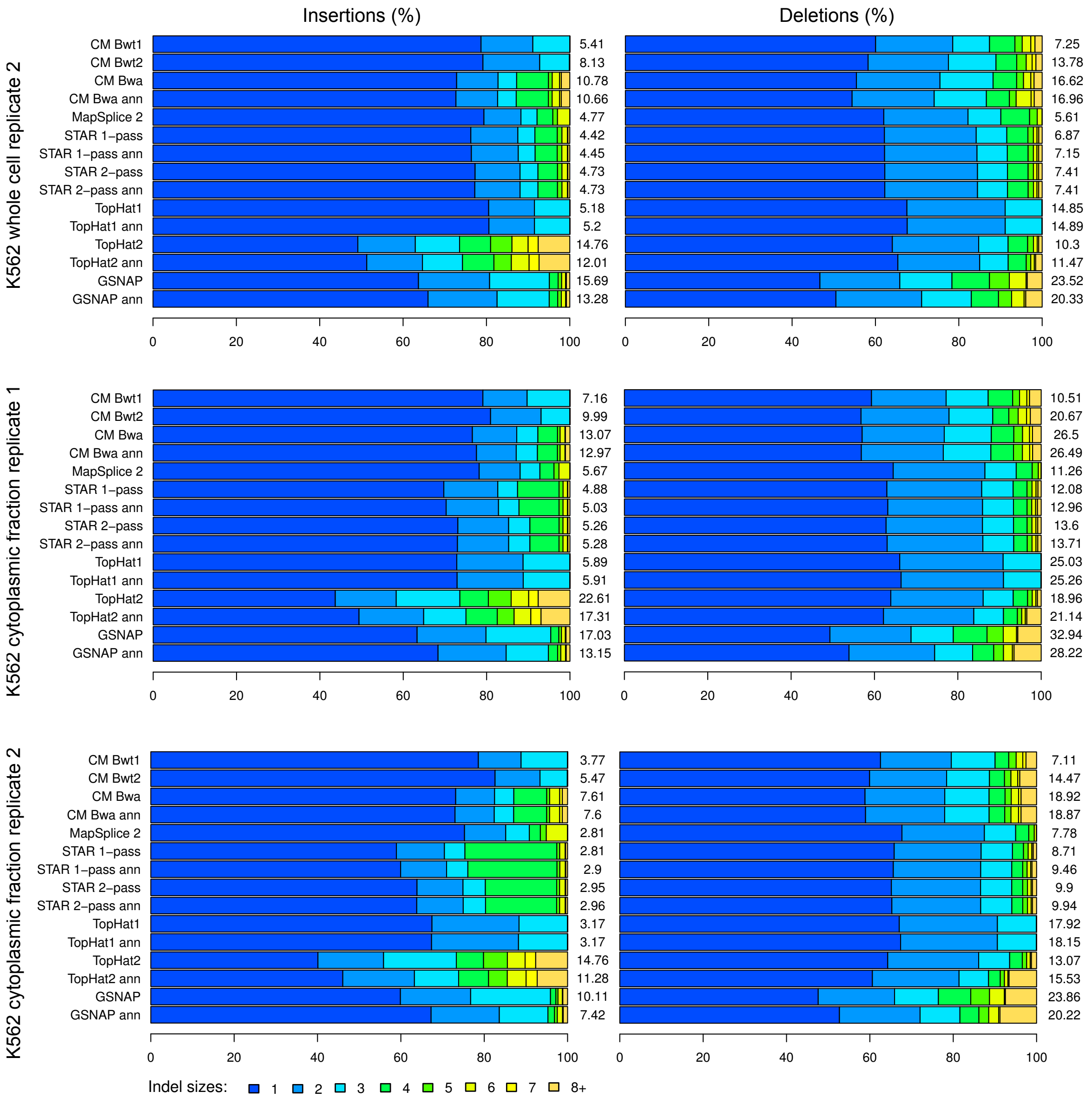

Supplementary Figure 9 Fraction of mapped reads with different indel sizes among all reads with indels for the second replicate of the K562 whole cell sample and both replicates of the K562 cytoplasmic fraction sample. Numbers next to the barplots indicate the number of mapped reads with indels divided by 10^5 (i.e. number of reads per 100,000).

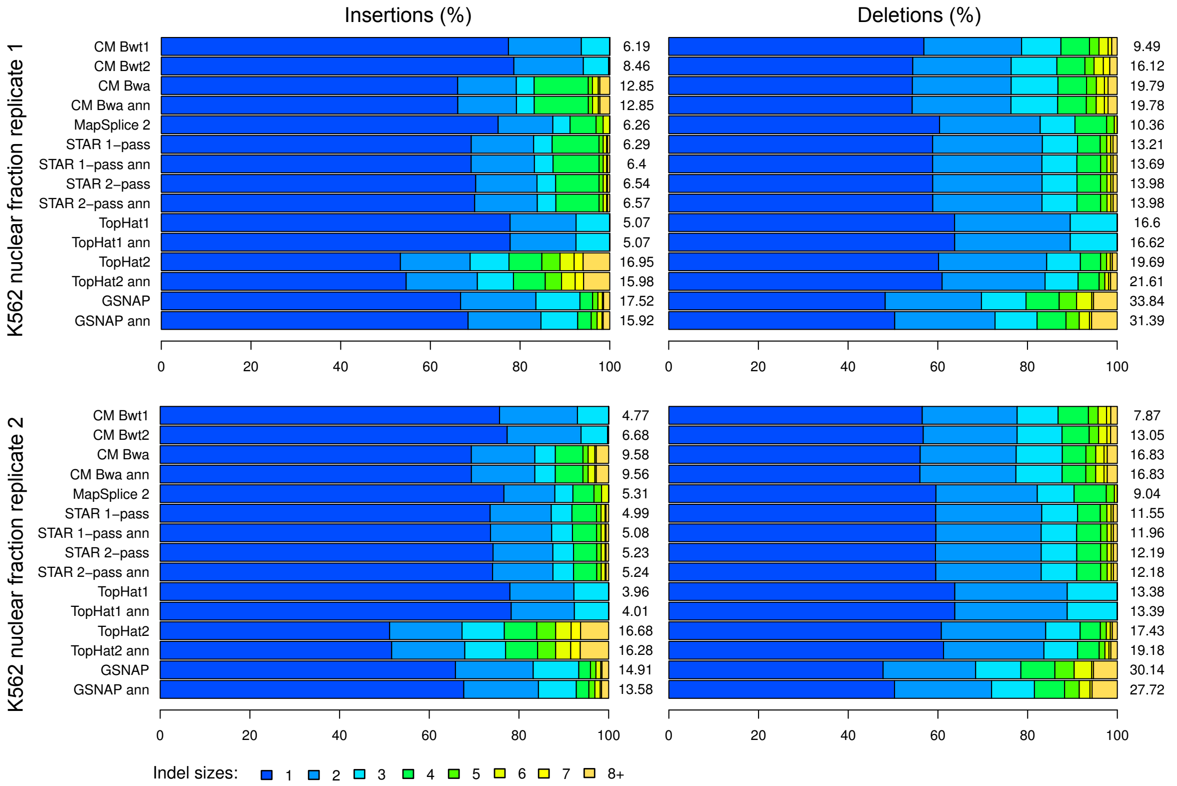

Supplementary Figure 10 Fraction of mapped reads with different indel sizes among all reads with indels for both replicates of the K562 nuclear fraction sample. Numbers next to the barplots indicate the number of mapped reads with indels divided by 10^5 (i.e. number of reads per 100,000).

Supplementary Tables

Dataset	ID	# Sequenced fragments	# Reads
Simulation 1	NA	40000000	80000000
Simulation 2	NA	40000000	80000000
K562 whole cell replicate 1	LID16627	113588758	227177516
K562 whole cell replicate 2	LID16628	119053315	238106630
K562 cytoplasmic fraction replicate 1	LID8465	124826068	249652136
K562 cytoplasmic fraction replicate 2	LID8466	88445339	176890678
K562 nuclear fraction replicate 1	LID8556	117113622	234227244
K562 nuclear fraction replicate 2	LID8557	105769104	211538208

Supplementary Table 1 Data sets used for evaluation and number of sequenced fragments and reads for each data set.

Program	Uniquely mapped reads					All reads (primary alignments only)				
	Overall mapped reads	Perfectly mapped reads	Part correctly mapped	Correctly mapped bases	Incorrectly mapped bases	Overall mapped reads	Perfectly mapped reads	Part correctly mapped	Correctly mapped bases	Incorrectly mapped bases
**A Simulation 1 All Reads**										
CM Bwt1	89.28	87.82	0.60	88.38	0.90	89.28	87.82	0.60	88.38	0.90
CM Bwt2 ($k = 3$, default)	95.12	93.44	0.57	93.97	1.15	95.12	93.44	0.57	93.97	1.15
CM Bwt2 ($k = 10$)	95.78	94.45	0.58	95.00	0.78	95.78	94.45	0.58	95.00	0.78
CM Bwa	95.99	94.68	0.55	95.20	0.79	95.99	94.68	0.55	95.20	0.79
CM Bwa ann	96.24	94.95	0.54	95.46	0.78	96.24	94.95	0.54	95.46	0.78
MapSplice 2	96.54	93.47	2.49	95.52	0.61	98.89	94.78	2.52	96.86	1.61
STAR 1-pass	96.14	84.61	11.20	94.87	0.47	98.72	85.81	11.35	96.20	1.70
STAR 1-pass ann	95.56	88.83	6.60	95.06	0.16	98.81	90.64	6.94	97.19	1.27
STAR 2-pass	95.48	89.11	6.24	95.01	0.16	98.82	91.05	6.57	97.26	1.23
STAR 2-pass ann	95.29	89.00	6.16	94.84	0.15	98.81	91.08	6.52	97.26	1.25
TopHat1	93.37	90.12	1.96	92.00	1.37	95.23	90.87	2.00	92.79	2.44
TopHat1 ann	93.51	90.21	2.00	92.13	1.37	95.39	90.97	2.05	92.94	2.45
TopHat2	91.38	90.41	0.46	90.84	0.54	93.81	91.45	0.56	91.96	1.85
TopHat2 ann	92.00	91.35	0.36	91.69	0.31	94.62	92.64	0.54	93.16	1.46
GSNAP	95.65	82.99	12.39	94.68	0.37	99.23	84.90	12.66	96.84	1.75
GSNAP ann	95.72	87.04	8.62	95.30	0.07	99.24	89.07	8.82	97.52	1.35
**B Simulation 2 All Reads**										
CM Bwt1	80.59	78.74	0.95	79.64	0.95	80.59	78.74	0.95	79.64	0.95
CM Bwt2 ($k = 3$, default)	86.38	84.08	0.98	85.01	1.37	86.38	84.08	0.98	85.01	1.37
CM Bwt2 ($k = 10$)	87.39	85.30	1.00	86.25	1.13	87.39	85.30	1.00	86.25	1.13
CM Bwa	87.91	86.20	0.92	87.07	0.84	87.91	86.20	0.92	87.07	0.84
CM Bwa ann	88.28	86.58	0.91	87.44	0.83	88.28	86.58	0.91	87.44	0.83
MapSplice 2	93.53	85.58	7.03	90.77	0.94	95.93	86.97	7.13	92.23	1.84
STAR 1-pass	93.36	72.55	20.39	90.75	0.62	96.23	73.72	20.74	92.21	1.96
STAR 1-pass ann	93.33	76.53	16.55	91.66	0.36	96.71	78.10	17.11	93.73	1.60
STAR 2-pass	93.24	76.80	16.14	91.58	0.39	96.77	78.54	16.74	93.85	1.58
STAR 2-pass ann	93.08	76.85	15.98	91.51	0.35	96.77	78.67	16.61	93.90	1.59
TopHat1	83.98	80.94	2.04	82.90	1.08	86.09	81.76	2.14	83.82	2.27
TopHat1 ann	84.40	81.32	2.08	83.32	1.07	86.53	82.16	2.19	84.26	2.27
TopHat2	75.53	74.31	0.87	75.13	0.40	77.92	75.29	0.97	76.18	1.74
TopHat2 ann	77.05	75.94	0.83	76.73	0.32	79.65	77.14	1.02	78.10	1.55
GSNAP	94.28	70.94	22.95	92.48	0.47	97.95	72.54	23.45	94.55	2.01
GSNAP ann	94.31	74.66	19.48	93.17	0.19	97.97	76.35	19.91	95.27	1.70

Supplementary Table 2 Fraction [in %] of overall mapped reads, perfectly mapped reads, part correctly mapped reads (of all simulated reads) as well as fraction of correctly and incorrectly mapped bases (of all bases in all simulated reads) on both simulated data sets. Results are shown separately for uniquely mapped reads and all mapped reads. In the latter case, only the primary alignment was evaluated. "CM Bwt1", "CM Bwt2" , "CM Bwa" denote ContextMap 2 used with Bowtie, Bowtie 2, and BWA as underlying alignment program, respectively. ContextMap 2 with Bowtie 2 was run with the maximum number of alignments reported per read (k) set to 3 (default setting used for evaluating mapping quality) and 10, respectively. If a gene annotation was provided, "ann" was added to the name of the respective program.

Program	Uniquely mapped reads					All reads (primary alignments only)				
	Overall mapped reads	Perfectly mapped reads	Part correctly mapped	Correctly mapped bases	Incorrectly mapped bases	Overall mapped reads	Perfectly mapped reads	Part correctly mapped	Correctly mapped bases	Incorrectly mapped bases
**A Simulation 1 Unspliced Reads**										
CM Bwt1	89.99	89.19	0.11	89.29	0.70	89.99	89.19	0.11	89.29	0.70
CM Bwt2 ($k = 3$, default)	95.65	94.49	0.11	94.58	1.07	95.65	94.49	0.11	94.58	1.07
CM Bwt2 ($k = 10$)	96.26	95.37	0.13	95.48	0.78	96.26	95.37	0.13	95.48	0.78
CM Bwa	96.45	95.59	0.12	95.69	0.76	96.45	95.59	0.12	95.69	0.76
CM Bwa ann	96.48	95.59	0.15	95.72	0.75	96.48	95.59	0.15	95.72	0.75
MapSplice 2	96.32	95.28	0.74	95.81	0.30	99.05	96.86	0.75	97.40	1.43
STAR 1-pass	96.01	90.69	5.17	95.68	0.15	98.70	92.04	5.25	97.10	1.41
STAR 1-pass ann	95.76	90.43	5.22	95.46	0.12	98.73	92.08	5.33	97.21	1.32
STAR 2-pass	95.73	90.42	5.21	95.45	0.10	98.73	92.11	5.33	97.24	1.30
STAR 2-pass ann	95.65	90.29	5.24	95.34	0.12	98.73	92.04	5.37	97.21	1.33
TopHat1	93.76	92.87	0.13	93.00	0.77	95.77	93.70	0.14	93.83	1.94
TopHat1 ann	93.76	92.86	0.13	92.99	0.77	95.77	93.70	0.14	93.83	1.94
TopHat2	92.20	91.78	0.14	91.92	0.29	94.73	92.89	0.15	93.03	1.70
TopHat2 ann	92.19	91.67	0.19	91.85	0.34	94.65	92.85	0.25	93.08	1.57
GSNAP	95.46	87.50	7.85	95.03	0.11	99.21	89.64	8.03	97.35	1.52
GSNAP ann	95.45	87.51	7.89	95.08	0.05	99.21	89.68	8.09	97.44	1.42
**B Simulation 2 Unspliced Reads**										
CM Bwt1	83.86	82.70	0.38	83.06	0.80	83.86	82.70	0.38	83.06	0.80
CM Bwt2 ($k = 3$, default)	88.16	86.48	0.40	86.86	1.30	88.16	86.48	0.40	86.86	1.30
CM Bwt2 ($k = 10$)	89.25	87.72	0.41	88.10	1.15	89.25	87.72	0.41	88.10	1.15
CM Bwa	89.50	88.36	0.39	88.72	0.78	89.50	88.36	0.39	88.72	0.78
CM Bwa ann	89.53	88.37	0.41	88.75	0.78	89.53	88.37	0.41	88.75	0.78
MapSplice 2	94.19	89.61	4.06	92.49	0.52	97.01	91.31	4.13	94.25	1.54
STAR 1-pass	93.74	79.84	13.68	92.64	0.26	96.65	81.13	13.90	94.14	1.64
STAR 1-pass ann	93.66	79.71	13.74	92.55	0.25	96.77	81.20	14.02	94.30	1.58
STAR 2-pass	93.56	79.59	13.76	92.45	0.26	96.79	81.18	14.07	94.33	1.57
STAR 2-pass ann	93.51	79.52	13.77	92.39	0.26	96.79	81.14	14.09	94.31	1.59
TopHat1	85.55	84.41	0.59	84.97	0.58	87.77	85.29	0.63	85.89	1.88
TopHat1 ann	85.56	84.41	0.59	84.98	0.58	87.78	85.29	0.63	85.90	1.87
TopHat2	77.90	77.20	0.51	77.69	0.21	80.44	78.25	0.54	78.76	1.68
TopHat2 ann	78.01	77.15	0.55	77.68	0.33	80.41	78.22	0.62	78.80	1.61
GSNAP	94.29	75.62	18.47	93.21	0.18	98.10	77.39	18.89	95.38	1.77
GSNAP ann	94.28	75.64	18.50	93.26	0.13	98.10	77.42	18.93	95.44	1.71

Supplementary Table 3 Fraction [in %] of overall mapped reads, perfectly mapped reads, part correctly mapped reads (of all simulated *unspliced* reads) as well as fraction of correctly and incorrectly mapped bases (of all bases in all simulated *unspliced* reads) on both simulated data sets. Results are shown separately for uniquely mapped reads and all mapped reads. In the latter case, only the primary alignment was evaluated. "CM Bwt1", "CM Bwt2" , "CM Bwa" denote ContextMap 2 used with Bowtie, Bowtie 2, and BWA as underlying alignment program, respectively. ContextMap 2 with Bowtie 2 was run with the maximum number of alignments reported per read (k) set to 3 (default setting used for evaluating mapping quality) and 10, respectively. If a gene annotation was provided, "ann" was added to the name of the respective program.

Program	Uniquely mapped reads					All reads (primary alignments only)				
	Overall mapped reads	Perfectly mapped reads	Part correctly mapped	Correctly mapped bases	Incorrectly mapped bases	Overall mapped reads	Perfectly mapped reads	Part correctly mapped	Correctly mapped bases	Incorrectly mapped bases
**A Simulation 1 Spliced Reads**										
CM Bwt1	86.40	82.22	2.58	84.69	1.71	86.40	82.22	2.58	84.69	1.71
CM Bwt2 ($k = 3$, default)	92.96	89.13	2.43	91.45	1.51	92.96	89.13	2.43	91.45	1.51
CM Bwt2 ($k = 10$)	93.82	90.71	2.44	93.04	0.78	93.82	90.71	2.44	93.04	0.78
CM Bwa	94.09	90.99	2.28	93.17	0.92	94.09	90.99	2.28	93.17	0.92
CM Bwa ann	95.27	92.32	2.13	94.38	0.89	95.27	92.32	2.13	94.38	0.89
MapSplice 2	97.42	86.04	9.68	94.36	1.88	98.23	86.27	9.77	94.66	2.37
STAR 1-pass	96.68	59.73	35.86	91.57	1.79	98.81	60.31	36.32	92.53	2.88
STAR 1-pass ann	94.73	82.28	12.26	93.43	0.35	99.14	84.77	13.53	97.11	1.03
STAR 2-pass	94.46	83.70	10.47	93.22	0.41	99.18	86.72	11.66	97.34	0.95
STAR 2-pass ann	93.82	83.73	9.90	92.80	0.29	99.16	87.13	11.23	97.46	0.93
TopHat1	91.77	78.88	9.43	87.93	3.84	93.03	79.29	9.64	88.53	4.50
TopHat1 ann	92.48	79.36	9.66	88.63	3.85	93.81	79.82	9.89	89.30	4.51
TopHat2	88.01	84.78	1.78	86.42	1.59	90.02	85.57	2.23	87.56	2.46
TopHat2 ann	91.24	90.04	1.08	91.06	0.18	94.51	91.82	1.75	93.47	1.04
GSNAP	96.44	64.51	31.00	93.21	1.44	99.31	65.51	31.61	94.78	2.68
GSNAP ann	96.82	85.14	11.58	96.20	0.18	99.36	86.57	11.81	97.84	1.07
**B Simulation 2 Spliced Reads**										
CM Bwt1	67.13	62.39	3.31	65.56	1.57	67.13	62.39	3.31	65.56	1.57
CM Bwt2 ($k = 3$, default)	79.05	74.17	3.38	77.39	1.65	79.05	74.17	3.38	77.39	1.65
CM Bwt2 ($k = 10$)	79.70	75.35	3.42	78.61	1.09	79.70	75.35	3.42	78.61	1.09
CM Bwa	81.33	77.29	3.11	80.27	1.06	81.33	77.29	3.11	80.27	1.06
CM Bwa ann	83.11	79.19	2.97	82.05	1.05	83.11	79.19	2.97	82.05	1.05
MapSplice 2	90.80	68.99	19.29	83.68	2.67	91.47	69.07	19.48	83.91	3.05
STAR 1-pass	91.80	42.50	48.05	82.96	2.14	94.50	43.11	48.93	84.26	3.30
STAR 1-pass ann	91.98	63.42	28.13	87.96	0.80	96.47	65.30	29.87	91.37	1.69
STAR 2-pass	91.89	65.27	25.99	87.96	0.95	96.70	67.61	27.80	91.90	1.62
STAR 2-pass ann	91.34	65.81	25.10	87.89	0.73	96.71	68.46	26.98	92.22	1.59
TopHat1	77.46	66.62	8.04	74.35	3.11	79.17	67.23	8.38	75.26	3.91
TopHat1 ann	79.59	68.59	8.23	76.49	3.10	81.39	69.26	8.60	77.50	3.90
TopHat2	65.76	62.38	2.35	64.57	1.19	67.56	63.07	2.73	65.54	2.02
TopHat2 ann	73.10	70.93	1.99	72.81	0.29	76.50	72.68	2.67	75.18	1.32
GSNAP	94.22	51.61	41.43	89.48	1.66	97.36	52.53	42.26	91.15	3.02
GSNAP ann	94.43	70.60	23.51	92.82	0.44	97.45	71.93	23.97	94.59	1.65

Supplementary Table 4 Fraction [in %] of overall mapped reads, perfectly mapped reads, part correctly mapped reads (of all simulated *spliced* reads) as well as fraction of correctly and incorrectly mapped bases (of all bases in all simulated *spliced* reads) on both simulated data sets. Results are shown separately for uniquely mapped reads and all mapped reads. In the latter case, only the primary alignment was evaluated. "CM Bwt1", "CM Bwt2" , "CM Bwa" denote ContextMap 2 used with Bowtie, Bowtie 2, and BWA as underlying alignment program, respectively. ContextMap 2 with Bowtie 2 was run with the maximum number of alignments reported per read (k) set to 3 (default setting used for evaluating mapping quality) and 10, respectively. If a gene annotation was provided, "ann" was added to the name of the respective program.

Program	Recall for simulated reads spanning different numbers of junctions					Precision for simulated reads spanning different numbers of junctions				
	1 (13808336)	2 (598297)	3 (11781)	2*(548382)	3*(6908)	1	2	3	2*	3*
**A Simulation 1**										
CM Bwt1	85.64	7.72	0.0	8.26	0.0	98.15	92.09	-	92.14	-
CM Bwt2	90.3	65.87	33.6	71.56	57.3	98.07	97.04	99.3	97.03	99.3
CM Bwa	91.94	72.05	36.44	78.27	62.13	98.34	97.12	99.4	97.09	99.37
CM Bwa ann	93.27	74.89	37.07	81.19	62.97	98.35	97.34	98.76	97.18	98.37
MapSplice 2	87.62	66.53	15.89	68.58	1.75	97.78	97.78	96.15	97.88	98.37
STAR 1-pass	65.28	18.47	2.58	19.18	0.75	95.74	94.68	87.86	94.89	94.55
STAR 1-pass ann	90.54	71.62	65.19	73.06	72.05	96.77	94.95	90.29	94.93	95.6
STAR 2-pass	92.41	76.57	71.54	78.23	78.58	97.74	96.91	96.23	96.94	98.19
STAR 2-pass ann	92.88	78.15	73.25	79.49	78.42	97.36	96.28	94.57	96.12	96.41
TopHat1	79.94	67.5	49.45	72.01	66.24	97.44	91.0	88.99	91.05	87.92
TopHat1 ann	80.41	70.21	55.28	73.63	67.49	97.23	90.27	88.77	90.16	87.22
TopHat2	86.13	78.64	63.27	83.07	80.2	98.06	97.35	98.07	97.39	98.75
TopHat2 ann	92.27	87.42	80.47	89.59	88.26	97.55	94.44	92.16	94.65	92.55
GSNAP	73.31	28.39	10.86	27.27	6.56	95.99	92.54	63.04	94.66	98.26
GSNAP ann	94.87	82.17	68.42	83.78	74.22	98.12	96.09	94.88	96.9	98.54
**B Simulation 2**										
	1 (14962090)	2 (622980)	3 (11701)	2* (509939)	3* (5602)	1	2	3	2*	3*
CM Bwt1	65.27	5.0	0.0	5.94	0.0	97.54	89.21	-	89.16	-
CM Bwt2	75.74	50.33	19.63	60.99	41.0	97.17	95.92	99.39	95.51	99.39
CM Bwa	78.63	57.26	20.96	69.35	43.75	97.77	96.28	98.71	95.79	98.63
CM Bwa ann	80.73	59.85	21.49	72.08	44.59	97.75	96.59	99.02	95.66	98.39
MapSplice 2	72.71	45.76	12.89	51.51	0.71	96.33	94.97	97.42	94.83	97.56
STAR 1-pass	51.7	11.13	0.63	12.73	0.34	94.78	93.35	89.16	92.83	70.37
STAR 1-pass ann	79.05	53.75	44.14	55.96	50.34	95.13	90.88	90.23	89.59	93.28
STAR 2-pass	81.47	59.59	47.77	63.35	56.19	95.87	92.55	92.05	91.45	94.11
STAR 2-pass ann	82.74	61.49	48.43	64.08	56.16	95.67	91.54	89.06	90.12	92.15
TopHat1	68.33	55.08	33.33	65.21	61.53	96.57	89.63	87.96	89.59	92.12
TopHat1 ann	70.45	58.86	44.22	67.64	63.39	96.58	88.91	88.37	88.47	89.72
TopHat2	64.11	56.92	38.39	65.37	60.55	96.96	95.63	95.37	95.62	95.36
TopHat2 ann	73.8	68.66	64.37	71.27	73.97	96.37	91.22	88.94	91.42	91.34
GSNAP	68.15	23.82	8.97	22.19	3.86	95.39	86.77	82.22	93.58	96.0
GSNAP ann	90.99	76.41	56.16	78.55	60.96	97.41	92.81	88.85	94.33	96.09

Supplementary Table 5 Recall and precision [in %] for spliced reads with different number of spanned junctions for simulation 1 and 2. Columns marked with an asterisk show results only for reads for which all exons except the first and last exon had length $\geq$ 20 nt.

Program	Recall for individual insertion lengths								Precision for individual insertion lengths							
	1	2	3	4	5	6	7	8	1	2	3	4	5	6	7	8
**Simulation 1 Insertions**																
CM Bwt1	56.6	60.0	57.0	0.0	0.0	0.0	0.0	0.0	96.9	97.7	93.7	-	-	-	-	-
CM Bwt2	72.0	77.8	74.1	1.6	0.5	0.0	0.0	0.0	97.0	98.2	93.3	69.6	30.7	0.0	-	0.0
CM Bwa	78.9	83.5	82.7	69.9	70.3	60.1	73.4	64.1	97.9	98.3	95.8	92.0	92.5	92.4	93.7	37.0
CM Bwa ann	79.2	83.7	83.0	69.9	70.3	60.1	73.4	64.1	98.0	98.4	96.0	92.0	92.4	92.4	93.6	37.2
MapSplice 2	73.7	60.4	59.8	55.1	50.7	39.7	0.0	0.0	97.4	98.2	97.0	93.1	94.8	90.6	-	-
STAR 1-pass	49.8	49.5	48.0	45.6	40.4	26.8	40.8	31.5	97.6	98.4	97.1	96.2	97.5	99.1	98.5	95.6
STAR 1-pass ann	55.8	54.5	51.7	47.4	51.9	28.5	41.0	41.8	97.9	98.3	96.8	96.1	96.5	99.1	98.6	96.6
STAR 2-pass	54.4	53.8	51.2	47.3	51.3	27.7	41.1	41.8	97.8	98.4	97.2	96.3	97.9	82.7	98.5	91.9
STAR 2-pass ann	56.1	54.9	51.9	47.8	51.9	28.7	41.1	41.8	97.9	98.3	96.8	96.1	97.9	99.1	98.5	96.6
TopHat1	65.9	72.7	72.2	0.0	0.0	0.0	0.0	0.0	94.8	96.2	90.3	-	-	-	-	-
TopHat1 ann	68.8	76.0	74.9	0.0	0.0	0.0	0.0	0.0	94.9	96.4	90.7	-	-	-	-	-
TopHat2	70.5	72.5	69.6	65.9	64.0	55.0	70.0	52.4	88.0	89.7	76.8	63.5	38.2	18.3	21.4	1.1
TopHat2 ann	84.8	86.0	84.8	82.7	86.8	85.9	86.4	90.1	94.0	94.1	85.9	81.3	65.2	41.5	43.5	4.2
GSNAP	85.2	87.2	85.8	53.9	54.2	49.6	54.7	54.6	91.1	88.1	67.8	87.9	93.7	62.7	86.2	18.0
GSNAP ann	87.0	88.5	87.2	53.9	54.9	50.7	55.3	54.9	95.5	95.7	82.7	89.2	92.9	67.7	93.9	52.1

**Simulation 1 Deletions**	Recall for individual deletion lengths								Precision for individual deletion lengths							
	1	2	3	4	5	6	7	8	1	2	3	4	5	6	7	8
CM Bwt1	61.2	60.1	58.0	54.1	69.7	43.5	51.1	50.3	97.7	97.1	91.8	95.3	92.2	60.8	92.4	84.9
CM Bwt2	74.8	73.3	73.3	64.1	80.6	68.3	52.9	61.0	97.7	97.3	93.9	87.2	93.4	85.0	81.4	93.1
CM Bwa	78.9	76.7	79.1	67.5	84.8	73.6	51.3	66.2	98.3	97.9	94.9	95.0	93.0	77.8	82.8	79.4
CM Bwa ann	79.3	77.2	79.4	69.4	85.0	73.6	51.3	67.2	98.3	98.1	95.0	96.4	93.1	77.9	92.2	79.6
MapSplice 2	77.9	76.0	79.9	68.0	83.3	63.7	0.0	0.0	98.4	98.7	94.9	96.4	99.3	99.9	-	-
STAR 1-pass	57.1	56.2	59.6	47.0	54.9	43.1	28.7	29.9	97.9	97.3	94.9	96.2	95.3	96.2	91.8	66.4
STAR 1-pass ann	64.5	62.5	65.0	53.7	57.9	43.8	29.1	30.8	98.4	96.5	96.5	92.3	94.6	97.8	96.9	55.7
STAR 2-pass	63.2	60.2	64.0	52.5	55.9	43.8	29.1	30.2	98.6	97.6	96.6	96.2	95.3	97.6	92.0	55.6
STAR 2-pass ann	64.9	62.8	65.7	54.2	57.9	43.8	29.1	30.8	98.5	96.6	96.6	92.3	94.6	97.8	96.9	55.7
TopHat1	69.7	69.2	71.0	0.0	0.0	0.0	0.0	0.0	95.3	96.2	92.6	-	-	-	-	-
TopHat1 ann	72.6	72.6	73.7	0.0	0.0	0.0	0.0	0.0	95.4	96.4	92.8	-	-	-	-	-
TopHat2	70.4	68.6	67.4	57.0	65.3	43.4	53.8	31.9	93.1	95.6	92.8	93.6	89.8	84.0	87.0	83.8
TopHat2 ann	84.0	82.0	81.3	73.6	81.5	66.9	72.9	37.9	95.7	96.3	94.2	91.6	93.5	77.7	93.8	64.3
GSNAP	86.5	83.8	85.9	78.9	81.9	76.2	68.9	70.3	92.4	87.7	71.0	47.7	24.7	9.8	74.3	57.0
GSNAP ann	87.7	85.8	87.0	82.7	83.3	78.3	84.6	74.1	96.2	93.9	87.6	85.3	66.8	48.1	89.7	63.6

Supplementary Table 6 Recall and precision for insertions and deletions in simulation 1.

Program	Recall for individual insertion lengths								Precision for individual insertion lengths							
	1	2	3	4	5	6	7	8	1	2	3	4	5	6	7	8
**Simulation 2 Insertions**																
CM Bwt1	41.7	41.9	41.9	0.0	0.0	0.0	0.0	0.0	95.8	96.2	93.6	-	-	-	-	-
CM Bwt2	58.3	58.1	59.7	0.1	0.1	0.0	0.0	0.2	96.2	96.3	94.2	63.7	94.9	0.0	0.0	44.4
CM Bwa	65.0	65.5	66.6	61.2	62.1	43.6	64.6	61.8	97.1	97.3	95.1	94.7	96.1	81.1	85.8	63.2
CM Bwa ann	65.2	65.7	66.8	61.4	67.6	44.2	64.7	61.8	97.1	97.3	95.1	94.6	96.4	81.3	85.8	65.6
MapSplice 2	54.6	42.8	42.7	38.3	42.9	33.2	0.0	0.0	95.5	96.6	96.0	94.4	94.5	85.3	-	-
STAR 1-pass	39.4	35.8	35.2	31.8	37.5	26.1	33.9	20.8	95.3	93.8	93.0	91.6	92.8	63.1	93.6	55.8
STAR 1-pass ann	44.9	40.4	39.3	34.6	38.3	28.7	38.6	23.1	95.8	94.1	92.4	91.7	92.4	55.6	93.7	55.6
STAR 2-pass	44.3	40.2	39.2	34.2	38.3	31.2	38.2	23.0	95.8	94.3	92.5	91.9	92.3	60.2	93.7	53.8
STAR 2-pass ann	45.4	41.0	40.0	34.9	38.3	28.9	39.0	23.1	95.9	94.1	92.5	91.7	92.3	55.7	93.7	54.9
TopHat1	52.2	54.6	54.0	0.0	0.0	0.0	0.0	0.0	93.0	94.9	93.3	-	-	-	-	-
TopHat1 ann	54.7	57.3	56.3	0.0	0.0	0.0	0.0	0.0	93.3	95.1	93.5	-	-	-	-	-
TopHat2	55.4	54.5	53.9	48.2	58.9	47.4	48.2	49.1	92.5	92.4	89.2	84.3	85.7	43.3	31.8	6.3
TopHat2 ann	67.9	67.8	68.2	69.6	67.9	62.1	67.6	61.1	94.6	94.7	92.7	89.4	91.2	55.3	49.5	7.5
GSNAP	78.3	78.8	78.9	41.7	48.2	43.1	47.3	38.5	93.2	92.8	87.5	93.8	96.5	76.5	84.2	50.1
GSNAP ann	80.0	80.9	81.4	42.1	48.3	43.7	47.7	38.1	94.1	94.1	91.0	93.1	96.8	77.8	90.0	62.6
**Simulation 2 Deletions**	Recall for individual deletion lengths								Precision for individual deletion lengths							
	1	2	3	4	5	6	7	8	1	2	3	4	5	6	7	8
CM Bwt1	46.2	45.5	47.9	46.5	47.9	48.3	61.0	44.5	96.7	94.3	93.0	95.3	90.0	85.2	93.5	74.3
CM Bwt2	60.9	61.3	61.2	62.3	64.4	63.7	73.0	58.0	96.2	95.1	92.7	94.1	90.9	87.5	88.9	78.4
CM Bwa	66.4	66.0	67.1	64.7	66.9	70.5	76.6	59.9	97.4	95.7	94.3	95.8	90.7	88.2	92.6	64.6
CM Bwa ann	66.7	66.3	67.3	64.9	67.3	70.8	77.1	59.9	97.4	95.7	94.4	95.7	90.8	88.1	92.5	62.1
MapSplice 2	58.5	58.5	58.8	58.1	55.2	39.0	0.0	0.0	96.8	96.6	95.8	96.6	91.8	90.6	-	-
STAR 1-pass	45.0	44.3	44.3	43.3	38.3	34.6	36.0	31.9	95.7	94.3	91.5	92.5	87.3	89.2	90.1	88.3
STAR 1-pass ann	50.1	49.9	48.5	47.0	43.1	41.2	38.0	33.2	96.0	94.1	91.7	90.2	87.9	87.6	90.4	60.5
STAR 2-pass	49.3	49.1	48.1	46.4	40.9	39.6	36.1	33.2	96.1	94.2	91.7	91.1	87.8	88.7	90.0	87.9
STAR 2-pass ann	50.4	50.3	48.8	47.3	43.0	41.5	37.3	33.3	96.1	94.1	91.8	90.4	87.8	87.7	90.3	68.2
TopHat1	58.1	58.3	56.9	0.0	0.0	0.0	0.0	0.0	94.8	93.1	93.5	-	-	-	-	-
TopHat1 ann	60.4	60.9	59.3	0.0	0.0	0.0	0.0	0.0	94.8	93.3	93.7	-	-	-	-	-
TopHat2	56.1	54.6	51.2	48.8	42.8	43.7	46.9	37.9	93.8	94.1	94.2	93.7	91.2	91.8	95.7	85.0
TopHat2 ann	66.5	65.4	62.9	61.1	55.3	59.0	55.3	46.5	94.7	95.0	94.5	92.8	91.3	91.7	95.8	53.1
GSNAP	79.1	79.4	78.2	74.6	72.3	76.3	60.6	61.2	93.4	91.5	86.1	79.1	64.4	38.9	87.5	47.0
GSNAP ann	80.6	81.0	80.0	78.3	75.9	79.3	72.1	71.4	94.1	92.8	89.3	88.2	82.8	65.9	87.7	49.8

Supplementary Table 7 Recall and precision for insertions and deletions in simulation 2.
